# Updated penetrance estimates for recurrent copy number variants – an improved definition and formula

**DOI:** 10.1038/s41431-025-01948-0

**Published:** 2025-10-15

**Authors:** Shuxiang Goh, Tracy Dudding-Byth, Mark Pinese, Edwin P. Kirk

**Affiliations:** 1 School of Women and Children’s Health, University of New South Wales, Sydney, NSW Australia; 2 New South Wales Health, Sydney, NSW Australia; 3 Genetics of Learning Disability, Newcastle, NSW Australia; 4 University of Newcastle, Newcastle, NSW Australia; 5 Children’s Cancer Institute, Sydney, NSW Australia; 6 Centre for Clinical Genetics, Sydney Children’s Hospital, Sydney, NSW Australia; 7 NSW Health Pathology Randwick Genomics Laboratory, Sydney, NSW Australia

**Keywords:** Cytogenetics, Clinical genetics, Neurodevelopmental disorders

## Abstract

Many copy number variants (CNVs) are implicated in neurodevelopmental disability, but exhibit incomplete penetrance. The definition of penetrance is often unclear. In published literature, penetrance typically includes the background risk of disease, while clinicians tend to exclude risks unrelated to the genetic variant. We propose a more clinically relevant definition of penetrance and develop a new formula for this. These changes are applied to existing data sources to produce updated penetrance estimates. Our findings indicate that most CNVs studied have significantly lower penetrance than previously published. Eleven CNVs, previously described as low-penetrant, are recalculated as having a penetrance close to 0% for intellectual disability. These include 1q21.1 proximal duplications [*RBM8A*], 2q11.2 deletions [*TMEM127*], 2q13 proximal deletions and duplications [*NPHP1*], 6q16 duplications [*SIM1*], 13q12 deletions [*CRYL1*], 15q11.2 duplications [*NIPA1*, *NIPA2*], 15q13.3 duplications [*CHRNA7*], 16p12.2 duplications [*CDR2*], 16p13.11 duplications [*MYH11*] and Xp22.3 duplications [*SHOX*]. Previous estimates of CNV penetrance, which ranged from 10–40% have been recalculated as 1–10%. In conclusion, many previously published estimates of CNV penetrance are inflated. Re-evaluation of existing data reveals lower and more accurate penetrance estimates for intellectual disability. This has important implications for diagnosis, genetic counselling, and prenatal reporting of recurrent CNVs.

## Introduction

Penetrance describes how often a genetic change leads to a specific phenotype. Penetrance is 100% or complete if the genetic change always results in a phenotype. If the phenotype does not always occur, it is termed incomplete penetrance. A non-pathogenic variant should be 0% penetrant. We demonstrate that published estimates of penetrance do not reflect this when using a common Bayesian formula for penetrance. We present a revised definition of penetrance that is more clinically useful, along with a new mathematical formula to estimate penetrance. These updated concepts apply to all forms of genetic penetrance, including copy number variations (CNVs), single-nucleotide variants, and other genetic changes.

Although the exact wording may vary between sources, the commonly published definition of penetrance is [1–6]:

*The probability of manifesting a phenotype given that a specific genetic variant is present*.

In large studies, this is generally [3–5, 7–15] calculated as follows:

Formula 1 $${\rm{Penetrance}}=P\left(D|G\right)=\frac{P\left({G|D}\right).P\left(D\right)}{P\left(G\right)}=\frac{P\left({G|D}\right).P\left(D\right)}{P\left({G|D}\right).P\left(D\right)+P\left({G|}{D}^{c}\right).P\left({D}^{c}\right)}$$

where P(D) is the probability of manifesting the phenotype (**D**isease) (equivalently, the prevalence of the phenotype in the general population), P(G) is the probability of having the genetic change (**G**enotype), P(D|G) is the probability of manifesting the phenotype given that the genotype is present, P(G|D) is the probability of having the genotype given that the phenotype is present, D^c^ refers to not having the phenotype and P(G|D^c^) is the probability of having the genotype given that the phenotype is absent.

However, in clinical practice, most clinicians interpret penetrance as the probability that a genetic variant *causes* a specific phenotype. These represent two different definitions of penetrance, as the latter excludes individuals who have the phenotype due to unrelated causes.

Two studies [8, 12] recognised this distinction and provided an alternate penetrance estimate that attempted to exclude individuals that incidentally shared the phenotype by subtracting the value P(D) from Formula 1. We build on this approach by developing Formula 2, which is more mathematically precise, to achieve the same outcome.

Penetrance estimates using Formula 1 [3, 4, 7, 9–11, 13–17] or Formula 2 (below) to estimate penetrance are most accurate when considering penetrance for a single pathology. Penetrance for multiple phenotypes is not supported by the underlying mathematical framework of the formulas (Supplementary Method 3.1). Given that the most common phenotype associated with recurrent CNVs is intellectual disability (ID) or developmental delay [18], this study focuses on CNV penetrance for ID.

This study addresses six key issues that lead to more accurate penetrance estimates:

1. Clarification of the definition of penetrance.
2. An updated formula reflecting the clarified definition.
3. Limiting penetrance estimations to a single phenotype of ID.
4. Determining the prevalence of ID.
5. Combining these changes to determine updated ID penetrance estimates for recurrent CNVs.
6. Updating 95% confidence intervals for penetrance estimates.

The net outcome is improved penetrance estimates for recurrent CNVs, with many CNVs demonstrating a lower penetrance for ID than previously published.

## Methods

We propose the following **new definition of penetrance**:

*Penetrance of a genetic variant is the probability of manifesting a phenotype due to having the genetic variant*.

The new definition differs from the earlier definition by excluding phenotypes incidental to the genetic variant, with concrete examples provided in Supplementary Method 1.5. Penetrance is similar to, but distinct from, the attributable fraction among the exposed, or the risk difference, which are commonly seen in other areas of statistics and medicine (Supplementary Method 1.4).

A mathematical formula for calculating penetrance can be developed using two different (but equivalent) approaches. Both methods begin by recognising that penetrance is the probability of having a phenotype given the presence of a specific genetic change, minus the probability that the phenotype is present due to another unrelated condition. Both approaches also take into consideration the possibility of dual diagnoses that may contribute to the phenotype. The two methods use different approaches (Supplementary Methods 1.1–1.4), but resultin the same formula:

Formula 2 $${\rm{Penetrance}}=\frac{P\left(D\right)}{P\left(G\right)}\,.\,\frac{P\left({G|D}\right)\,-\,P\left(G\right)}{1-\,P\left(G\right)-P\left(D\right)+P\left(D\right).P\left({G|D}\right)}$$

P(D), P(G) and P(G|D) were described previously.

It should be noted that the data required to calculate either the earlier (Formula 1) or new (Formula 2) version of penetrance are the same. Therefore, data used to derive penetrance estimates using the earlier formula can be used with this new formula – no additional data is required to calculate updated penetrance estimates.

Most individuals in affected cohorts [3, 4, 7, 10, 14, 15, 17, 19] selected for this study were children from high-income countries with ID or developmental delay [3, 4, 7, 10, 14, 15, 17]. Therefore, the parameter P(D) in Formula 1 and Formula 2, most accurately refers to the prevalence of paediatric ID in high-income countries. This was taken to be 1.1% based on data from a systematic review [20] (Supplementary Methods 3.2–3.3).

The inclusion criteria for CNVs in Table 1 were (i) availability of published penetrance estimates for the CNV, (ii) The ability to verify these estimates using supplied data, (iii) ID and/or developmental delay being present in most of the affected cohort (or can be assumed to be present – see Supplementary Methods 3.2) and (iv) the CNV’s primary phenotype includes, or has been suggested to include ID.

These methods are similar to that of an earlier systematic review [21], so our data is similar, modified only to remove individuals without ID where possible (Supplementary Methods 2, 3.1, 3.2). Affected cohort data in this study is pooled from 8 studies [3, 4, 7, 10, 14, 15, 17, 19] that met the inclusion criteria listed above. The control cohort (*n* = 269,885) is a subset of gnomAD data which have been labelled by gnomAD as controls, and each one with a relevant CNV for this study was highlighted in the Supplemental in the systematic review [21].

Many methods for determining 95% confidence intervals were considered. Most [3, 4, 7, 9–11, 14, 15, 17, 19] rely on assumptions of normality and symmetry of data, which were not appropriate for rare CNVs due to small datasets. We applied a bootstrap method from an earlier study [21], modified by running the simulation 10,000 times instead (Code provided in Supplementary File).

## Results

A systematic review of ID [20] identified 17 studies reporting the prevalence of paediatric ID in high income countries. The prevalence ranged from 0.44 to 3.68% (Supplementary Methods 3.3), with a median prevalence of P(D) = 1.1% used in this study. Alternate methods for determining the optimal value of P(D) were considered (Supplementary Methods 3 & Supplementary Discussion 1) but were less accurate.

Table 1 reports updated penetrance estimates for ID using this new approach, alongside estimates using the earlier penetrance formula [21]. Data used to calculate these numbers are taken from a systematic review [21] and modified to remove individuals without ID where possible (Supplementary Methods 2). A larger version of this table considers penetrance estimates using other values of P(D) (Supplementary Results 1).

**Table 1:** Updated penetrance estimates of 83 recurrent CNVs.

Copy number variant	Size (Mb) criteria used in studies^d^	Deletion/Duplication	Pooled affected cohort	gnomAD controls	Penetrance using previous methods (95% confidence interval)^f^	Penetrance using new formula and 1.1% prevalence for ID (95% confidence interval)
1p36 deletion and 1p36 duplication [*GABRD*]^a^	10.0^c^	Del	78/32587	0/269885	100%^c^ (94–100)	100%^c^ (76–100)
		Dup	16/15767	0/269885	100%^c^ (84–100)	100%^c^ (52–100)
1q21.1 proximal deletion and 1q21.1 proximal duplication [*RBM8A*]^a^	c	Del	See Supplementary Table 4	See Supplementary Table 4	-	-
		Dup	85/48637	249/269885	9.2%^c^ (7.2–11)	1.0%^c^ (0.5–1.5)
1q21.1 distal deletion and 1q21.1 distal duplication [*GJA5*]^a^	0.82	Del	49/16190	71/269885	38% (30–47)	10% (7.1–15)
		Dup	28/16190	94/269885	21% (14–28)	4.2% (2.2–6.5)
2p16.3 deletion [*NRXN1*]^a^	c	Del	12/6623	45/269885	37%^c^ (22–51)	9.8%^c^ (4.4–17)
2q11.2 deletion [*TMEM127*]^a^	0.95	Del	2/15767	16/269885	10% ^NS^ (0–26)	1.2% ^NS^ (−1.1–5.9)
2q13 proximal deletion and 2q13 proximal duplication [*NPHP1*]^a^	0.16	Del	78/15767	1533/269885	4.5% ^NS^ (3.5–5.4)	−0.1% ^NS^ (−0.4 – 0.1)
		Dup	118/15767	1763/269885	5.8% ^NS^ (4.8–6.8)	0.2% ^NS^ (−0.1–0.4)
2q23.1 deletion [*MBD5*]	0.55^c^	Del	20/32587	0/269885	100% (78–100)	100% (42 – 100)
2q37 deletion and 2q37 duplication [*HDAC4*]^a^	2.8	Del	20/32587	0/269885	100%^c^ (78–100)	100%^c^ (42–100)
		Dup	2/32587	1/269885	47%^c, NS^ (0–100)	15%^c, NS^ (−1.1 –100)
3q29 deletion and 3q29 duplication [*DLG1*]^a^	1.6	Del	20/33010	6/269885	59%^c^ (39–84)	22%^c^ (11–52)
		Dup	18/32587	4/269885	67%^c^ (44–91)	29%^c^ (13–67)
4p16.3 deletion (Wolf-Hirschhorn Syndrome) and 4p16.3 duplication^a^	0.5	Del	17/32587	0/269885	100% (75–100)	100% (38–100)
		Dup	4/32587	3/269885	37% (8.1–100)	9.9% (0.7–100)
5q35.3 deletion (Sotos Syndrome) and 5q35.3 duplication [*NSD1*]^a^	1.4 or 1.7^c^	Del	14/32587	0/269885	100% (69–100)	100% (31–100)
		Dup	4/32587	0/269885	100%^c^ (31–100)	100%^c^ (7.4–100)
6p25 deletion and 6p25 duplication	5.9	Del	See Supplementary Table 4	See Supplementary Table 4	-	-
		Dup	See Supplementary Table 4	See Supplementary Table 4	-	-
6q16 deletion and 6q16 duplication [*SIM1*]^a^	0.07^c^	Del	1/23380	3/269885	17% ^NS^ (0–100)	3.0% ^NS^ (−1.1–100)
		Dup	1/23380	5/269885	11% ^NS^ (0–38)	1.4% ^NS^ (−1.1–10)
7q11.23 deletion (Williams-Beuren Syndrome) and 7q11.23 duplication^a^	1.4	Del	84/33010	2/269885	95%^c^ (87–100)	79%^c^ (59–100)
		Dup	41/33010	7/269885	72%^c^ (56–89)	34%^c^ (20–62)
8p23.1 deletion and 8p23.1 duplication [*CLDN23*, *SOX7*, *GATA4*]^a^	3.8	Del	See Supplementary Table 4	See Supplementary Table 4	-	-
		Dup	24/32587	0/269885	100%^c^ (81–100)	100%^c^ (47–100)
9q34 deletion (Kleefstra syndrome) and duplication [*EHMT1*]^a^	3.3	Del	18/32587	0/269885	100% (75–100)	100% (38–100)
		Dup	8/32587	0/269885	100%^c^ (55–100)	100%^c^ (19–100)
10q23 deletion and 10q23 duplication [*NRG3*, *GRID1*, *BMPR1A*]^a^	6.8 or 7.1 or 7.2^c^	Del	See Supplementary Table 4	See Supplementary Table 4	-	-
		Dup	5/32587	6/269885	27%^c^ (7.2–59)	6.1%^c^ (0.5–22)
13q12 deletion [*CRYL1*]^a^	0.2^c^	Del	14/15767	227/269885	5.3% ^NS^ (2.8–8.3)	0.1% ^NS^ (−0.5–0.8)
15q11.2 deletion [BP1-BP2] and 15q11.2 duplication [*NIPA1*, *NIPA2*]^a^	0.2 or 0.25 or 0.29 or 0.5^c^	Del	312/40561	956/269885	10% (9.3–12)	1.3% (1.0–1.6)
		Dup	149/31215	1331/269885	4.9% ^NS^ (4.2–5.7)	0% ^NS^ (−0.2–0.1)
15q11.2 deletion [BP1-BP3] and 15q11.2 duplication [*NIPA1*, *NIPA2*] (Prader-Willi Syndrome/Angelman Syndrome)^a^	5.4 or 5.5^c^	Del	4/423	0/269885	100% (97–100)	100% (88–100)
		Dup	54/51418	11/269885	58%^c^ (44 – 76)	21%^c^ (13 – 39)
15q11q13 deletion [BP2-BP3] (Prader-Willi Syndrome/Angelman Syndrome) and 15q11.13 duplication^a^	3.6	Del	60/32587	0/269885	100% (92–100)	100% (70–100)
		Dup	82/32587	11/269885	77%^c^ (66–88)	40%^c^ (28–60)
15q13.3 deletion [BP4-BP5] and 15q13.3 duplication [*CHRNA7*]^a^	1.31 or 1.35 or 1.49^c^	Del	87/33010	27/269885	59%^c^ (48–69)	22%^c^ (15–31)
		Dup	27/32587	140/269885	7.9%^c^ (5.1–11)	0.7%^c^ (0.0001–1.4)
15q13.3 smaller deletion and 15q13.3 smaller duplication [*CHRNA7* and *OTUD7A* only]^a^	0.44^c^	Del	c	c	c	c
		Dup	c	c	c	c
15q24 deletion and 15q24 duplication [*BBS4*, *PML*, *SIN3A*]^a^	1.5 or 2.53 or 3.01^c^	Del	10/33010	0/269885	100% (60–100)	100% (23–100)
		Dup	4/32587	3/269885	37%^c^ (6.9–100)	9.9%^c^ (0.4–100)
15q24.2q24.5 deletion and 15q24.2q24.5 duplication [*FBXO22*, *TSPAN3*]^a^	1.79 or 2.23	Del	5/32587	1/269885	69%^c^ (29–100)	31%^c^ (6.6–100)
		Dup	6/32587	1/269885	73%^c^ (31–100)	35%^c^ (7.4–100)
15q25.2 proximal deletion and 15q25.2 proximal duplication [*RPS17*, *HOMER2*, *BNC1*]^a^	1.56	Del	See Supplementary Table 4	See Supplementary Table 4	-	-
		Dup	4/23380	1/269885	71%^c^ (24–100)	33%^c^ (5.0–100)
16p13.3 deletion (Rubinstein-Taybi Syndrome) [*CREBBP*]^a^	0.1	Del	10/32587	1/269885	82%^c, b^ (51–100)	47%^c, b^ (17–100)
16p13.11 deletion and 16p13.11 duplication [*MYH11*]^K^	0.16–2.62^c^	Del	See Supplementary Table 4	See Supplementary Table 4	-	-
		Dup	143/48600	398/269885	9.6%^c^ (8.0–11)	1.1%^c^ (0.7–1.5)
16p12.2 deletion (previously 16p12.1 deletion) and 16p12.2 duplication (previously 16p12.1 duplication) [*CDR2*]^a^	0.42 or 0.52 or 0.55^c^	Del	62/33226	139/269885	16%^c^ (12–20)	2.8%^c^ (1.8–4.0)
		Dup	16/32587	135/269885	5.0% ^NS^ (2.7–7.6)	0% ^NS^ (−0.5–0.6)
16p11.2p12.2 deletion (previously 16p11.2p12.1) and 16p11.2p12.2duplication (previously 16p11.2p12.1 duplication)^a^	7.57	Del	20/32587	0/269885	100% (78–100)	100% (42–100)
		Dup	14/32587	0/269885	100% (71–100)	100% (33–100)
16p11.2 distal deletion and 16p11.2 distal duplication [*SH2B1*]^a^	0.23^c^	Del	46/33649	32/269885	38%^c^ (28–50)	10%^c^ (6.6–16)
		Dup	35/33226	81/269885	16%^c^ (11–22)	2.7%^c^ (1.4–4.4)
16p11.2 proximal deletion and 16p11.2 proximal duplication [*TBX6*]^a^	0.55 or 0.65^c^	Del	155/33649	58/269885	53%^c^ (46–61)	18%^c^ (14–24)
		Dup	95/33649	73/269885	36% (29–43)	9.4% (6.9–13)
17p13.3 deletion and 17p13.3 duplication [*YWHAE*]^a^	0.05 or 0.8^c^	Del	7/32587	0/269885	100% (47–100)	100% (15–100)
	Dup	6/15767	8/269885	41%^c^ (15–69)	12%^c^ (2.5–31)	
17p13.3 deletion and 17p13.3 duplication [*PAFAH1B1*]	0.1 or 0.56^c^	Del	8/32587	0/269885	100% (53–100)	100% (18–100)
		Dup	4/15767	1/269885	79% (31–100)	43%^c^ (7.7–100)
17p12 deletion (Hereditary Neuropathy with Liability to Pressure Palsies) and 17p12 duplication (Charcot Marie Tooth) [*PMP22*]^a^	1.33 or 1.43	Del	3/15767	146/269885	1.8% ^NS^ (0–4.2)	−0.7%^NS^ (−1.1–−0.2)
		Dup	9/16190	75/269885	9.7% ^NS^ (4.2–16)	1.1%^NS^ (−0.2–2.8)
17p11.2 deletion (Smith-Magenis Syndrome) and 17p11.2 duplication (Potocki-Lupski Syndrome) [*RAI1*]^a^	1.46 or 3.39^c^	Del	35/33010	0/269885	100% (86–100)	100% (56–-100)
		Dup	25/32587	1/269885	92%^c^ (75–100)	69%^c^ (38–100)
17q11.2 deletion (Neurofibromatosis Type 1) and 17q11.2 duplication [*NF1*]^a^	1.2	Del	26/33010	2/269885	85%^c^ (66–100)	54%^c^ (28–100)
		Dup	35/33010	4/269885	79%^c^ (63–95)	44%^c^ (25–79)
17q12 deletion (Renal Cysts and Diabetes) and 17q12 duplication [*HNF1B*]^a^	1.26 or 1.39 or 1.51	Del	See Supplementary Table 4	See Supplementary Table 4	-	-
		Dup	38/33649	61/269885	21% (15–28)	4.2% (2.4–6.6)
17q21.31 deletion (Koolen-de Vries Syndrome) and 17q21.31 duplication [*MAPT*, *KANSL1*]^a^	0.17 or 0.48^c^	Del	42/32587	0/269885	100% (89–100)	100% (62–100)
		Dup	5/32587	1/269885	69% (30–100)	31% (7.0–100)
17q23 deletion and 17q23 duplication [*TBX2*, *TBX4*]	2.04 or 2.1	Del	6/32587	0/269885	100% (47–100)	100% (15–100)
		Dup	1/32587	1/269885	31%^c, NS^ (0–100)	7.4%^c, NS^ (−1.1 –100)
19p13.12 deletion	3.62	Del	13/32587	0/269885	100% (69–100)	100% (31–100)
22q11.2 deletion Velocardiofacial Syndrome and 22q11.2 duplication [*TBX1*]^a^	1.24 or 2.88^c^	Del	188/33010	10/269885	89%^c^ (83–95)	63%^c^ (50–80)
		Dup	146/49060	167/269885	20%^c^ (17–24)	4.0%^c^ (3.0–5.2)
22q11.2 distal deletion and 22q11.2 distal duplication [*BCR*, *MAPK1*]^a^	1.75 or 1.82 or 1.87	Del	26/23803	1/269885	94% (81–100)	76% (46–100)
		Dup	18/23803	7/269885	61%^c^ (41–83)	24%^c^ (12–50)
22q13.33 deletion Phelan-McDermid Syndrome and 22q13.33 duplication [*SHANK3*]^a^	0.06^c^	Del	45/15767	3/269885	93%^c, b^ (85–100)	74%^c, b^ (53–100)
		Dup	c	c	c	c
Xp22.3 duplication [*SHOX*]	0.04–1.3	Dup	83/18947	25/12594^e^	11%^e^ (7.2– 16)	1.3%^e^ (0.5–2.8)

Table adapted from a systematic review [21] showing genomic size and penetrance of 83 CNVs from 8 studies [3, 4, 7, 10, 14, 15, 17, 19]. When a CNV displays more than one size, the rationale for combining these as one CNV are discussed in the Supplementary Analysis in the systematic review [21]. Data from pooled affected and control cohorts are listed and may differ from that used in the systematic review due to this table being an estimate of penetrance for ID, with affected cohort data chosen to reflect individuals with ID when possible (Supplementary Methods 2). The studies that data were taken from and their genomic coordinates are shown in Supplementary Table 1. Penetrance is displayed in 2 columns – the first uses methodology employed by earlier studies and the second uses methodology from this study. Negative penetrance estimates can be considered a protective effect of the CNV (Supplementary Methods 1.6).

^a^: One or more studies publishing penetrance estimates for this CNV have been excluded, either because it is a duplicate dataset or for risk-of-bias reasons that are outlined in the systematic review from which this table was adapted [21].

^b^: This CNV is likely to be fully or nearly fully, penetrant for ID.

^c^: There are complexities in either the genomic coordinates or data used to calculate penetrance for this CNV. The listed penetrance estimate, both in this table and in Supplementary Table 4, can be misleading, and caution is recommended when interpreting these estimates in a clinical setting. A recent systematic review discussed the complexity of genomic coordinates or data for this CNV in their Supplementary Analysis [21].

^d^: Genomic coordinates are provided in Supplementary Table 1.

^e^: There is no gnomAD v4.0 CNV data for CNVs on the X chromosome. The original study’s control cohort is used instead for penetrance estimation.

^f^: Penetrance in this column is calculated using the pooled affected cohort and control data listed, along with the earlier formula for penetrance and 5.08% prevalence of disease. This method of calculation was outlined in a recent systematic review [21].

-: The affected cohort is composed predominantly of individuals with ID, but the primary phenotype of this CNV is not ID. This means penetrance for ID cannot be calculated for this CNV using this data. (Supplementary Methods 3 & Supplementary Results 1).

^NS^: This penetrance estimate is not statistically significant. Statistical significance is achieved when the lower 95% confidence interval is greater than 5.08% using the earlier penetrance formula or greater than 0% using the new penetrance formula. Negative penetrance estimates using the new formula occur when the prevalence of the CNV in control cohorts is greater than that in affected cohorts (Supplementary Methods 1.1).

Table 1 demonstrates that penetrance for ID for all CNVs are reduced, except for fully penetrant CNVs which remain fully penetrant. The difference is most noticeable for low penetrant CNVs, which demonstrate an approximate 5–10 fold reduction in penetrance estimates.

CNV penetrance estimates are most accurate when interpreted as penetrance for ID, rather than for any disease because (i) Most datasets used to calculate CNV penetrance are primarily comprised of individuals with ID (Supplementary Methods 3.2), (ii) ID is the primary phenotype associated with most recurrent CNVs [18] and (iii) the formula for estimating penetrance is less reliable when calculating multiple phenotypes combined together (Supplementary Methods 3.1). Some CNVs, such as the 17q12 [*HNF1B*] deletion, which causes renal cysts and diabetes, require a different affected cohort since ID is not prominently associated with the CNV. Penetrance estimates for these CNVs are excluded from Table 1, but provided in Supplementary Results 1 with additional notes.

Earlier penetrance estimates of 10%, 20% and 40% are re-estimated at 1%, 4% and 10% respectively. Fully penetrant conditions remain fully penetrant. A shortcut for converting penetrance estimates from earlier publications to the new penetrance is provided in Supplementary Results 3.

It is useful to note that penetrance for ID in the 17p12 [*PMP22*] deletion for Hereditary Neuropathy with Liability to Pressure Palsies and the reciprocal duplication for Charcot Marie Tooth Disease is 0%. This is a correct result, as these conditions are not associated with ID [22, 23]. This also provides supporting evidence that the datasets predominantly contain patients with ID rather than phenotypes that might be associated with these two conditions.

## Discussion

Using a revised penetrance formula with an improved understanding of its parameters resulted in lower penetrance estimates for many CNVs compared to previous publications [3, 4, 7, 10, 14, 15, 17, 19, 21]. Reduced CNV penetrance estimates may affect clinical management of patients with these CNVs. Reduced CNV penetrance estimates for ID that are close to 0% suggest minimal or non-pathogenicity for ID.

### Improvements to penetrance estimations

Our study provides more accurate penetrance estimates due to two key improvements built on prior work [5, 14, 24] in this field.

The first improvement involves clarifying the definition of penetrance, so that background risk is excluded. Formula 1 (based on the earlier definition) includes the possibility that the genetic change is present but unrelated to the phenotype. Formula 2 (based on the newly proposed definition) excludes the background rate of this phenotype. When CNV prevalence is similar in both affected and control groups, Formula 1 will report 1–6% penetrance whilst Formula 2 will report 0%. Stated in another manner, Formula 1 erroneously adds the chance that the control cohort has the phenotype, whilst Formula 2 excludes this.

The second improvement involves a recognition that neither Formula 1 nor 2 are suited to calculate penetrance for multiple phenotypes (Supplementary Methods 3.1). Whilst an ideal study should focus on a single well-defined condition (such as ID), most studies [3, 4, 7, 10, 14, 15, 17, 19] include participants with a variety of conditions (such as ID, developmental delay, autism, and congenital malformations). Nonetheless, if most individuals have ID (or developmental delay which is often an early sign of ID), then penetrance for ID can still be approximated. The prevalence of ID, P(D) was determined from a systematic review to be approximately 1.1% (Supplementary Methods 3 & Supplementary Discussion 1). Using this instead of P(D) = 3% [9], 4% [4, 10, 11, 15], 5.12% [7, 12, 14], 5.3% [7, 12, 14], or 13% [19], led to an estimated 3–12 fold reduction in penetrance estimates for ID, compared to penetrance estimates previously published.

This change in lowering the value of P(D) to 1.1% requires justification. The affected cohort of three studies were clearly comprised entirely of individuals with ID (or infants with developmental delay) [4, 17, 19], justifying the use of 1.1% for these studies. In other studies [3, 7, 10, 14, 15], affected cohorts were comprised mostly of individuals with ID. Supplementary Methods 3 justifies using these datasets to approximate the penetrance of ID. Supplementary Discussion 1 explores alternate approaches to penetrance estimation, before concluding on the approach presented here. The datasets are imperfect, so accurate penetrance estimates derived from these datasets are impossible. Within these constraints, the value of 1.1% is more accurate than using any other value.

### The phenotype associated with a CNV

Different CNVs may be associated with different phenotypes. However, the data sources used to calculate penetrance are the same regardless of the CNV. This has led to confusion regarding the phenotype a CNV might be penetrant for.

A key question arises as to whether the differences in penetrance estimates between this publication and earlier studies are due to a focus on ID rather than other phenotypes. We argue that for most CNVs, the penetrance listed for ID in Table 1 using the new method is likely more accurate and clinically-meaningful, regardless of this distinction. We expand on this below, with further details in Supplementary Discussion 1:

- For CNVs associated with a primary phenotype other than ID, penetrance estimates are omitted from Table 1. Examples include the 17q12 [*HNF1B*] deletion (Renal Cysts and Diabetes) and the 1q21.1 [*RBM8A*] proximal deletion. Neither penetrance for ID, nor penetrance for another phenotype, can be reliably estimated from these datasets. A different dataset could be used to estimate penetrance for the non-ID phenotype.
- For a CNV for which the primary phenotype is ID, the new method is clearly more accurate due to an updated formula and more appropriate choice of the parameter P(D) = 1.1% to represent the prevalence of ID. For this group, the presence or absence of other phenotypes for the CNV is irrelevant when it comes to choosing a value for P(D). This group constitutes the majority of the CNVs in Table 1.
- For CNVs that can cause ID in some individuals and congenital malformations without ID in other individuals, penetrance estimates presented here are still likely to be more accurate than previous estimates. This is because the affected cohort P(G | D) used to estimate penetrance of neurodevelopmental disorders and congenital malformations in CNVs are primarily composed of individuals with ID (Supplementary Methods 3.2), but also includes some individuals without ID. Therefore, the phenotype “D” is mix of individuals highly biased towards ID, supporting the use of 1.1% in the formula as the optimal parameter for the prevalence of the phenotype (Supplementary Methods 3).

Autism involves a non-homogenous mix of conditions with a definition that has changed significantly over time, whilst onset of schizophrenia is age-related. These issues make penetrance estimation using Formula 1 or Formula 2 challenging (Supplementary Methods 3.4).

Sometimes, the phenotype thought to be associated with a CNV may be incidental and its description in relation to the CNV may be due to ascertainment bias. Consider a hypothetical CNV with 10% penetrance for ID (and therefore 90% of individuals with this CNV are unaffected). Multiple authors have assumed a background chance of disability being approximately 5% [3, 4, 7, 10, 11, 14, 15, 17]. Under these assumptions, 4.5% (5% of 90%) will have a different disability due to causes unrelated to the CNV (incidental phenotypes). Individuals with this CNV may therefore have ID (10%), other disabilities (4.5%) or be healthy (85.5%). Case studies are biased towards those with a phenotype, so case studies may describe other disabilities in up to 30% (4.5/(10 + 4.5)%) of individuals with this CNV. This is higher than what most would consider as the background risk and can be mistaken for being part of the phenotype, but is clearly just an aspect of ascertainment bias. This theoretical example demonstrates how incidental pathology can be misattributed to low penetrant CNVs (with a rate of up to 30% in this example) due purely to ascertainment bias, when in reality, penetrance of these pathologies should be 0%.

### Examples of changes to CNV penetrance

Seven CNVs are recalculated to have 0% penetrance for ID, or have 95% confidence intervals which include 0%. These are 0.9 Mb 2q11.2 [*TMEM127*] deletions, 0.2 Mb 2q13 [*NPHP1*] proximal deletions and duplications, 0.13 Mb 6q16 [*SIM1*] duplications, 0.2 Mb 13q12 [*CRYL1*] deletions, 0.3 Mb 15q11.2 [BP1-BP2] [*NIPA1*, *NIPA2*] duplications and 0.5 Mb 16p12.2 [*CDR2*] duplications (previously mismapped to 16p12.1). The prevalence of these CNVs in affected and control cohorts are similar. These 7 CNVs account for 1.8% of individuals in gnomAD controls (Supplementary Result 2).

Four CNVs are calculated to have approximately 1% penetrance for ID and 95% confidence intervals that include values ≤ 1%. These are 0.3 Mb 1q21.1 [*RBM8A*] proximal duplications, 1.4 Mb 15q13.3 [BP4-BP5] [*CHRNA7*] duplications,1 Mb 16p13.11 [*MYH11*] duplications and Xp22.3 [*SHOX*] duplications. The 95% confidence intervals for these do not include 0% penetrance, suggesting pathogenicity and low (but statistically significant) penetrance. However, it is suggested that these results be interpreted conservatively, as incomplete penetrance may not be the correct explanation for the slight difference in prevalence of this CNV in affected compared to control datasets. Differences in genetic ancestry [25], publication bias and other confounding variables in the way control and affected cohorts are selected may provide alternate explanations [21]. These four CNVs account for 0.5% of individuals in gnomAD controls (Supplementary Results 2).

Three CNVs with 1–3% penetrance for ID may have unclear pathogenicity using these methods. The slight enrichment of these CNVs in affected compared to control cohorts may represent susceptibility to ID, but could also represent bias or noise in the dataset. This may differ from CNV to CNV. These CNVs are 0.3 Mb 15q11.2 [BP1-BP2] [*NIPA1*, *NIPA2*] deletions, 0.5 Mb 16p12.2 deletions [*CDR2*] (previously mismapped to 16p12.1) and 0.2–0.3 Mb 16p11.2 distal duplications [*SH2B1*]. Phenotypes associated with these CNVs have been published in case series of affected individuals. However, given the high prevalence of individuals with these variants in gnomAD controls, it’s likely that many individuals with these CNVs are either healthy or have features that lie within a spectrum of human normal. The earlier example of an incidental phenotype with up to 30% penetrance being erroneously associated with a CNV, demonstrates how ascertainment bias can lead to misattribution of pathogenicity, and is particularly relevant for CNVs in this group. These 3 CNVs account for 0.4% of individuals in gnomAD controls (Supplementary Results 2).

Some recurrent CNVs are rare and data is sparse. CNVs that are present in very few individuals can result in wide 95% confidence intervals for penetrance that span from 0 to 100%. Penetrance for these CNVs using these methods are uncertain. More broadly, for any CNV, it may be more appropriate to interpret currently available data for penetrance as lying within a range of potential penetrance estimates (e.g., a 95% confidence interval), rather than as a fixed percentage (Supplementary Discussion 2).

### Implications for clinical care

These findings have multiple clinical implications. Fourteen CNVs with penetrance close to 0% are seen in approximately 2.7% of individuals labelled by gnomAD as controls. In contrast, approximately 3.1% of gnomAD controls harbour at least one of the 83 CNVs in Table 1 (Supplementary Results 2). Equivalently, 14/83 CNVs in Table 1 with little evidence for pathogenicity for ID or other phenotypes make up the vast majority of recurrent CNVs in gnomAD controls with published penetrance estimates, whilst the majority 69/83 of recurrent CNVs that are more penetrant are seen in only a minority of gnomAD controls. Choosing not to report these 14 CNVs will dramatically reduce the number of healthy individuals who are given a pathogenic CNV result, and dramatically reduce laboratory and clinician workload. Reporting these 14 CNVs as pathogenic poses a risk of misattributing a patient’s phenotype to the CNV, or mislabelling a healthy individual. Moreover, if a diagnosis is based on one of these CNVs, it may reduce the likelihood of the patient being offered further genomic testing. Consequently, the practice of reporting these 14 common CNVs may compromise patient care by limiting a more comprehensive diagnostic evaluation in some countries or settings.

Large scale studies of some of these CNVs demonstrate that on average, people with some of these 14 CNVs may perform poorer in some cognitive tasks than those without [11]. However, it is unclear if all such individuals perform poorer, or if some perform poorly and others perform normally, which would also lead to similar graphs in studies that aggregate the results. Given how common these 14 CNVs are, the lack of strong correlation with a non-intellectual phenotype and the inability to prove that they cause cognitive reduction in all individuals, it is worth considering that reporting it as pathogenic in an individual may be an example of false positive reporting for that individual. Given that normal human intellect spans a large range from IQ 70–130 and that there are likely many polygenic factors that contribute to this variable range, it may be more helpful to consider these 14 CNVs as part of a normal spectrum of human genetic changes rather than as pathogenic.

A less stringent cut-off could consider 11 CNVs (seen in 2.3% of gnomAD controls) instead of 14 CNVs (Supplementary Result 2).

In the prenatal setting, identifying a CNV previously reported as being 5–10% penetrant and recognising that an accurate measure of penetrance is closer to 0%, may make the difference between a laboratory choosing to report the CNV or not to report. The choice not to report such CNVs, as suggested by some prenatal reporting guidelines [12, 26], can make a significant difference to anxiety and the choices families make in pregnancy. Choosing not to report these CNVs can also improve the quality of life for families, by reducing their time spent performing unnecessary research on the CNV.

### Observations from other studies that are consistent with findings from this study

Our findings have the potential to resolve conflicting observations in the literature regarding pathogenicity of very low penetrant CNVs. For example, the 15q13.3 duplication [*CHRNA7*] [BP4-BP5] has been reported as pathogenic [4, 27, 28] and as having little evidence for pathogenicity [29]. This study clarifies the paradox as being due to the misleading definition of penetrance and resolves it with Formula 2.

Many recent studies suggest caution when assigning pathogenicity to low-penetrant CNVs, especially in the prenatal setting [12, 26, 30]. For example, it has been suggested that the 16p11.2 distal duplication [*SH2B1*] be not reportable in the prenatal setting [26], despite its penetrance of 16% [21] which intuitively appears high enough to warrant reporting. Revised penetrance estimates in this study show that penetrance for this CNV is closer to 2.7% (95% CI 1.4–4.4%). Our study also raises the possibility that this apparent positive 2.7% penetrance estimate, could be due to other factors such as differences in genetic ancestry or imperfectly ascertained cohorts. Therefore, this study provides a mathematical justification for some current published guidelines regarding not reporting low penetrant CNVs.

Some prospective studies of neonates with CNVs [31, 32] demonstrate less evidence of neurodevelopmental disability on follow-up than one might expect based on earlier penetrance estimates. The lower penetrance estimates in this study provide a mathematical reason why this might be so.

Additionally, it is common in clinical practice to see many low penetrant CNVs being inherited from unaffected parents. This observation can be explained by the lower penetrance estimates published in this study.

### Limitations in estimating penetrance using the Bayesian formula

Penetrance estimates for CNVs provided in this study should be interpreted cautiously. The listed penetrance estimates are for ID (because the data sources are biased towards ID) rather than for penetrance of other phenotypes. Pleiotropy of CNVs means some CNVs might have a higher penetrance for other phenotypes. Accurate penetrance estimates also rely on large datasets containing affected and control individuals. While differences in CNV prevalence between these two groups are typically interpreted as evidence for incomplete penetrance of CNVs [3–5, 7–12, 14–17, 19], other factors may explain these differences. These include cohort selection biases, age-related onset of symptoms, variable expressivity and methodological differences.

In particular, the genetic makeup of affected and control cohorts can be challenging to compare because affected cohorts are typically recruited from one or two hospitals or geographic locations [3, 4, 10, 14, 15, 17] whilst control cohorts are often drawn from diverse studies and locations [3, 4, 10, 14, 33]. This disparity raises the possibility that observed increases in CNV prevalence in affected cohorts could stem from differences in genetic ancestry or geography [25], rather than pathogenicity. The 3q29 [*DLG1*] [21], 15q11.2 [*NIPA1*, *NIPA2*] [25], and 15q13.3 [*CHRNA7*] [BP4-BP5] [21, 25] CNVs may represent examples of this.

Penetrance estimates in this study are based on data that has been collected imperfectly. Control cohorts are commonly comprised of adults from outpatient clinics who were enroled as affected individuals for various (non-neurodevelopmental) health issues such as cancer [3, 4, 14], hyperlipidemia [3, 14], atherosclerosis [14], diabetes [15] or asthma [3, 14]. Therefore, control cohorts may include those with comorbid ID. This can result in fully penetrant conditions being misinterpreted as incompletely penetrant. Table 1 flags, with the symbol ‘b’, several CNVs in this category. The opposite may also be true of affected cohorts, particularly if there is no clinical information on the pathology request form for microarray.

Technological differences between the way affected and control cohorts are identified may also affect penetrance estimates. Most CNVs are likely to be detected equally well using either microarray assemblies or CNV callers for exome data [34]. However, some CNVs may be identified by one method and not the other. If so, then a disparity in prevalence between affected cohorts (which use microarrays) versus the gnomAD control cohort (which uses exomes) may lead to erroneously higher or lower penetrance estimates for that particular CNV.

Despite these potential confounding factors, a significant enrichment of a recurrent CNV in affected compared to control cohorts provides reasonable evidence for pathogenicity, though caution is advised as the aforementioned factors may cause over- or under-estimation of the true penetrance.

Finally, there is a blurry line between concepts of incomplete penetrance and variable expressivity. A CNV may reduce cognitive performance in some areas, but be insufficient to cause intellectual disability [11]. In this sense, calling the CNV non-penetrant for ID could be misleading, as it does have an effect. On the other hand, if the effect size is small and the individual does not consider themselves impaired, it may be kinder in clinical practice to refer to their ability as lying within a spectrum of human normal, rather than focussing their attention on merely one of many polygenic factors affecting intellect.

## Conclusion

We propose an updated definition and formula for genetic penetrance that excludes the background rate of disease. If a genetic change does not predispose to a genetic illness, this genetic change should be considered 0% penetrant for that illness. Previous definitions and formulas always result in penetrance >0% due to inclusion of the background risk, which could be misleading for clinicians and patients.

Imprecise penetrance estimates have the potential to be detrimental by limiting diagnostic options for affected individuals. There is potential for harm in classifying low penetrant CNVs as “pathogenic”, when most individuals are either unaffected or so mildly affected that their symptoms fall within a spectrum of human normal. The updated penetrance estimates presented here may affect interpretation and reporting of CNVs, with implications for genetic counselling in both prenatal and postnatal settings.

## Supplementary information

Supplementary Materials

Supplementary Table 6 & 7

Penetrance calculation

## Data Availability

All relevant data are listed within the paper and its supporting information files (Supplementary Discussion 1 & Supplementary Results 1).

## Code availability

Penetrance estimates and their 95% confidence interval can be obtained by running Bootstrap_Penetrance.m in Octave v8.3.0.

## References

[1] Carruth ED, Young W, Beer D, James CA, Calkins H, Jing L, et al. Prevalence aNd Electronic Health Record-based Phenotype Of Loss-of-function Genetic Variants in Arrhythmogenic Right Ventricular Cardiomyopathy-Associated Genes. Circ Genom Precis Med. 2019;12:e002579. 31638835 10.1161/CIRCGEN.119.002579 PMC6876858

[2] Chen S, Parmigiani G. Meta-analysis of BRCA1 and BRCA2 penetrance. J Clin Oncol. 2007;25:1329–33. 17416853 10.1200/JCO.2006.09.1066 PMC2267287

[3] Cooper DN, Krawczak M, Polychronakos C, Tyler-Smith C, Kehrer-Sawatzki H. Where genotype is not predictive of phenotype: Towards an understanding of the molecular basis of reduced penetrance in human inherited disease. Hum Genet. 2013;132:1077–130. 23820649 10.1007/s00439-013-1331-2 PMC3778950

[4] Kirov G, Rees E, Walters JT, Escott-Price V, Georgieva L, Richards AL, et al. The penetrance of copy number variations for schizophrenia and developmental delay. Biol Psychiatry. 2014;75:378–85. 23992924 10.1016/j.biopsych.2013.07.022 PMC4229045

[5] Vassos, Collier E, Holden DA, Patch S, Rujescu C, St D, et al. Penetrance for copy number variants associated with schizophrenia. Hum Mol Genet. 2010;19:3477–81. 20587603 10.1093/hmg/ddq259

[6] Roberts JD, Asaki SY, Mazzanti A, Bos JM, Tuleta I, Muir AR, et al. An International Multicenter Evaluation of Type 5 Long QT Syndrome: A Low Penetrant Primary Arrhythmic Condition. Circulation. 2020;141:429–39. 31941373 10.1161/CIRCULATIONAHA.119.043114 PMC7035205

[7] Allach El Khattabi L, Heide S, Caberg JH, Andrieux J, Doco Fenzy M, Vincent-Delorme C, et al. 16p13.11 microduplication in 45 new patients: refined clinical significance and genotype-phenotype correlations. J Med Genet. 2018;0:1–7. 10.1136/jmedgenet-2018-105389 30287593

[8] Chaste P, Sanders SJ, Mohan KN, Klei L, Song Y, Murtha MT, et al. Modest impact on risk for autism spectrum disorder of rare copy number variants at 15q11.2, specifically breakpoints 1 to 2. Autism Res. 2014;7:355–62. 24821083 10.1002/aur.1378 PMC6003409

[9] Cosemans N, Vandenhove L, Vogels A, Devriendt K, Van Esch H, Van Buggenhout G, et al. The clinical relevance of intragenic NRXN1 deletions. J Med Genet. 2020;57:347–55. 31932357 10.1136/jmedgenet-2019-106448

[10] Isles AR, Ingason A, Lowther C, Walters J, Gawlick M, Stober G, et al. Parental Origin Of Interstitial Duplications at 15q11.2-q13.3 in Schizophrenia And Neurodevelopmental Disorders. PLoS Genet. 2016;12:e1005993. 27153221 10.1371/journal.pgen.1005993 PMC4859484

[11] Kendall KM, Bracher-Smith M, Fitzpatrick H, Lynham A, Rees E, Escott-Price V, et al. Cognitive performance and functional outcomes of carriers of pathogenic copy number variants: analysis of the UK Biobank. Br J Psychiatry. 2019;214:297–304. 30767844 10.1192/bjp.2018.301 PMC6520248

[12] Maya I, Perlman S, Shohat M, Kahana S, YacobsonS, TenneT,et al. Should We Report 15q11.2 BP1-BP2 deletions and duplications in the prenatal setting?.J Clin Med.2020;9:11. 10.3390/jcm9082602 PMC7463673 32796639

[13] Mohan KN, Cao Y, Pham J, Cheung SW, Hoffner L, Ou ZZ, et al. Phenotypic association of 15q11.2 CNVs of the region of breakpoints 1-2 (BP1-BP2) in a large cohort of samples referred for genetic diagnosis. J Hum Genet. 2019;64:253–5. 30542208 10.1038/s10038-018-0543-7

[14] Rosenfeld JA, Coe BP, Eichler EE, Cuckle H, Shaffer LG. Estimates of penetrance for recurrent pathogenic copy-number variations. Genet Med. 2013;15:478–81. 23258348 10.1038/gim.2012.164 PMC3664238

[15] Tropeano M, Howley D, Gazzellone MJ, Wilson CE, Ahn JW, Stavropoulos DJ, et al. Microduplications at the pseudoautosomal SHOX locus in autism spectrum disorders and related neurodevelopmental conditions. J Med Genet. 2016;53:536–47. 27073233 10.1136/jmedgenet-2015-103621

[16] Al, Shehhi M, Forman EB, Fitzgerald JE, McInerney V, Krawczyk J, et al. NRXN1 deletion syndrome; phenotypic and penetrance data from 34 families. Eur J Med Genet. 2019;62:204–9. 30031152 10.1016/j.ejmg.2018.07.015

[17] Jønch AE, Douard E, Moreau C, Van Dijck A, Passeggeri M, Kooy F, et al. Estimating the effect size of the 15Q11.2 BP1-BP2 deletion and its contribution to neurodevelopmental symptoms: recommendations for practice. J Med Genet. 2019;56:701–10. 31451536 10.1136/jmedgenet-2018-105879 PMC6817694

[18] Unique. Rare Chromosome & Gene Disorder Guides: Rare Chromosome Disorder Support Group; 2024 Available from: https://rarechromo.org/disorder-guides/.

[19] Martin CL, Wain KE, Oetjens MT, Tolwinski K, Palen E, Hare-Harris A, et al. Identification of neuropsychiatric copy number variants in a health care system population. JAMA Psychiatry. 2020;77:1276–85. 32697297 10.1001/jamapsychiatry.2020.2159 PMC7376464

[20] Maulik PK, Mascarenhas MN, Mathers CD, Dua T, Saxena S. Prevalence of intellectual disability: a meta-analysis of population-based studies. Res Dev Disabil. 2011;32:419–36. 21236634 10.1016/j.ridd.2010.12.018

[21] Goh S, Thiyagarajan L, Dudding-Byth T, Pinese M, Kirk EP. A systematic review and pooled analysis of penetrance estimates of copy-number variants associated with neurodevelopment. Genet Med. 2025;27:101227. 39092588 10.1016/j.gim.2024.101227

[22] van Paassen BW, van der Kooi AJ, van Spaendonck-Zwarts KY, Verhamme C, Baas F, de Visser M. PMP22 related neuropathies: Charcot-Marie-Tooth disease type 1A and Hereditary Neuropathy with liability to Pressure Palsies. Orphanet J Rare Dis. 2014;9:38. 24646194 10.1186/1750-1172-9-38 PMC3994927

[23] Chrestian N Hereditary Neuropathy with Liability to Pressure Palsies. In: Adam MP, Feldman J, Mirzaa GM, Pagon RA, Wallace SE, Bean LJH, et al., editors. GeneReviews(®). Seattle (WA) 1993. 20301566

[24] Benn PA. Prenatal counseling and the detection of copy-number variants. Genet Med. 2013;15:316–7. 23552451 10.1038/gim.2013.16

[25] Schultz LM, Knighton A, Huguet G, Saci Z, Jean-Louis M, Mollon J, et al. Copy-number variants differ in frequency across genetic ancestry groups. HGG Adv. 2024;5:100340. 39138864 10.1016/j.xhgg.2024.100340 PMC11401192

[26] Muys J, Blaumeiser B, Jacquemyn Y, Bandelier C, Brison N, Bulk S, et al. The Belgian MicroArray Prenatal (BEMAPRE) database: A systematic nationwide repository of fetal genomic aberrations. Prenat Diagn. 2018;38:1120–8. 30334587 10.1002/pd.5373

[27] Zhou D, Gochman P, Broadnax DD, Rapoport JL, Ahn K. 15q13.3 duplication in two patients with childhood-onset schizophrenia. Am J Med Genet B Neuropsychiatr Genet. 2016;171:777–83. 26968334 10.1002/ajmg.b.32439 PMC5069586

[28] Gillentine MA, Schaaf CP. The human clinical phenotypes of altered CHRNA7 copy number. Biochem Pharmacol. 2015;97:352–62. 26095975 10.1016/j.bcp.2015.06.012 PMC4600432

[29] ClinGen. 15q13.3 recurrent region (D-CHRNA7 to BP5) (includes CHRNA7 and OTUD7A): ClinGen Dosage Sensitivity Curation Page; 2018 [updated 10/5/2018. Available from: https://dosage.clinicalgenome.org/clingen_region.cgi?id=ISCA-46295.

[30] Cai M, Que Y, Chen X, Chen Y, Liang B, Huang H, et al. 16p13.11 microdeletion/microduplication in fetuses: investigation of associated ultrasound phenotypes, genetic anomalies, and pregnancy outcome follow-up. BMC Pregnancy Childbirth. 2022;22:913. 36476185 10.1186/s12884-022-05267-w PMC9727942

[31] Muys J, Jacquemyn Y, Blaumeiser B, Bourlard L, Brison N, Bulk S, et al. Prenatally detected copy number variants in a national cohort: A postnatal follow-up study. Prenat Diagn. 2020;40:1272–83. 32436253 10.1002/pd.5751

[32] Smajlagić D, Lavrichenko K, Berland S, Helgeland Ø, Knudsen GP, Vaudel M, et al. Population prevalence and inheritance pattern of recurrent CNVs associated with neurodevelopmental disorders in 12,252 newborns and their parents. Eur J Hum Genet. 2021;29:205–15. 32778765 10.1038/s41431-020-00707-7 PMC7852900

[33] Fu J, Liao C, Collins R, Wang L, Ben-Isvy D, Brand H, et al. Rare coding CNVs from exome sequenced individuals in gnomAD v4 GnomAD: GnomAD; 2023 [updated 1/11/2023. Available from: https://gnomad.broadinstitute.org/news/2023-11-v4-copy-number-variants/.

[34] Babadi M, Fu JM, Lee SK, Smirnov AN, Gauthier LD, Walker M, et al. GATK-gCNV enables the discovery of rare copy number variants from exome sequencing data. Nat Genet. 2023;55:1589–97. 37604963 10.1038/s41588-023-01449-0 PMC10904014

