# Supplementary material for: Updated penetrance estimates for recurrent copy number variants – an improved definition and formula
Source: Eur J Hum Genet. 2025 Oct 15;34(1):119–27. doi: 10.1038/s41431-025-01948-0 (PMC12816643; doi:10.1038/s41431-025-01948-0)
Supplement: Supplementary file 1 — Supplementary Materials [file 41431_2025_1948_MOESM1_ESM.pdf]

Supplemental Materials overview

S. Methods 1 – Deriving the penetrance formula

- SM1.1 – Derivation of the newly proposed penetrance formula
- SM1.2 – One method of deriving the penetrance formula
- SM1.3 – A second method of deriving the penetrance formula
- SM1.4 – Similarity of this new penetrance formula to the attributable fraction among the exposed and risk difference
- SM1.5 – Comparison of earlier concepts and formula for penetrance to the new concept and formula
- SM1.6 – Negative penetrance estimates

S. Methods 2 – Studies used to estimate penetrance for CNVs associated with neurodevelopment

S. Methods 3 – An exploration of the terms P(D) and P(G|D) used in the formula

- SM3.1 – Importance for the penetrance formula to refer to a single phenotype
- SM3.2 – Composition of affected cohorts
- SM3.3 – Prevalence of intellectual disability
- SM3.4 – Penetrance for autism and schizophrenia

S. Results 1 – Supplementary table of penetrance estimates with different values of P(D)

S. Results 2 – Prevalence of very low penetrant CNVs in gnomAD v4.0 controls

S. Results 3 – Converting earlier penetrance estimates to new penetrance

S. Discussion 1 – Various methods of approaching the problem of penetrance using cohorts with multiple phenotypes

- S. Discussion 1.1 – How other studies approached the complexity of multiple phenotypes and limitations associated with these approaches
- S. Discussion 1.2 – A weighted value of P(D) for penetrance of multiple phenotypes
- S. Discussion 1.3 – Conclusion regarding the optimal value of P(D)

S. Discussion 2 – 95% confidence intervals for penetrance estimates

S. References

Supplemental Methods 1 – Deriving the penetrance formula

Supplemental Methods 1.1 – Derivation of the newly proposed penetrance formula

There are two (equivalent) methods of deriving the newly proposed penetrance formula. The first is by subtracting a scaled background rate, depending on how penetrant the genetic change is and taking into account the possibility of dual (or multiple) diagnoses. The second is by subtracting the background rate of disease and then scaling this value so that the range of penetrance outputs ranges from 0% to 100%. These two methods of deriving the penetrance formula can be thought of as two different ways, or two different methods of thinking, to arrive at the same endpoint.

For both methods, the underlying change is a new or clarified definition of penetrance. We propose a new (or clarified) definition of penetrance:

New definition: *Penetrance is the probability of manifesting a phenotype due to having a specific genetic change.*

This new definition avoids the mathematical ambiguity of the commonly-used definition, as discussed in the Introduction Section of the main paper.

Supplemental Methods 1.2 – One method of deriving the penetrance formula

This proposed new definition of penetrance can be developed into a mathematical formula by noting that penetrance is the probability of disease given a specific genetic change is present, minus the chance that the disease could be caused by something else, and taking into consideration the possibility of dual (or multiple) diagnoses. The following formula accomplishes this:

$$Penetrance = P(D|G) - \frac{1-P(D|G)}{1-P(D|G^c)} . P(D|G^c) \ , \qquad \text{(Formula 2a)}$$

where the terms are defined in the main text of this article, and again at the end of this section, for convenience.

The value (1-P(D|G))/(1-P(D|G^C)) in Formula 2a) will be referred to as the *weight*. This choice of weight satisfies all 3 scenarios that consider the possibility of a dual diagnosis:

- Fully penetrant genetic changes are considered under the scenario where P(D|G)=1. In this scenario, weight will be zero. Therefore, the subtraction term for the background rate of disease P(D|G^C) is zero. This is because the genetic change G is sufficient on its own to cause disease, even if there is a second reason for disease. Penetrance using Formula 2a is P(D|G) in this case.
- Non-penetrant (benign) genetic changes are considered under the scenario where P(D|G) = P(D|G^C). In this scenario, weight will be 1. Therefore, the subtraction term is the background rate of disease P(D|G^C). This is because if individuals with non-penetrant (or non-pathogenic) genetic changes are affected, then they are affected due to background reasons unrelated to their genetic change. Penetrance using the formula is 0% in this case.

- Genetic changes that are incompletely penetrant are considered under the scenario where P(D|G) takes a value between the two aforementioned possibilities. In this scenario, weight will take a value between 0 and 1. A highly penetrant and low penetrant example are given below:
    - Affected individuals with highly penetrant genetic changes are regarded as being penetrant (probably) because of their genetic condition with only a small subtraction of the background rate of disease. This small subtraction represents the few individuals in which this genetic change is not penetrant and their cause for disease can more correctly be attributed to background factors. Weight is small, and penetrance will be P(D|G) minus a small number.
    - Affected individuals with low penetrant genetic changes are regarded as being affected (probably) due to the background rate of disease and penetrance for these conditions need to take into account (i.e. subtract) the relatively greater role that the background rate contributes in this scenario. Weight is large (nearly 1) and penetrance will be P(D|G) – weight*P(D|G^C).

Taken in aggregate, this formulation of penetrance takes into account the possibility of dual (or multiple) diagnoses and reports a genetic change as being penetrant after removal of a weighted background rate that represents the chance the individual will still be affected even if they did not have another cause for their disease.

To algebraically reduce Formula 2a) to the equivalent Formula 2b) that was presented in the main text of this study

$$\text{Penetrance} = \frac{P(D)}{P(G)} \cdot \frac{P(G|D) - P(G)}{1 - P(G) - P(D) + P(D).P(G|D)} , \qquad \text{(Formula 2b)}$$

requires the following substitutions:

1. $P(G|D) = \frac{P(G).P(D|G)}{P(D)}$
2. $P(G^c) = 1 - P(G)$
3. $P(G^c|D) = 1 - P(G|D)$.

The terms in the formula are defined below:

- P(D) is the static parameter chosen to represent the prevalence of Disease in the population (e.g., 1.1% for intellectual disability in this study).
- P(G|D) is the prevalence of those with the Genetic variant in an affected cohort of individuals with Disease D.
- P(G) is the prevalence of the Genetic variant in the general population, which includes affected and unaffected individuals. P(G) is equal to P(G|D)*P(D) + P(G|D^c)*(1-P(D)), where P(G|D^c) is the prevalence of those with the genetic change in a cohort without disease (i.e. control cohort).
- P(D|G) and P(D|G^C) are the prevalences of the Disease in those with the Genetic variant and in those without the Genetic variant, respectively. Equivalently, they represent the probability that an individual has the Disease if they have and do not have the Genetic variant, respectively.

Supplemental Methods 1.3 – A second method of deriving the penetrance formula

There is an alternate (equivalent) way of deriving Formula 2.

Starting from the newly proposed definition in Supplemental Methods 1.1, it is noted that penetrance is the probability of manifesting the disease if you have a specific genetic change P(D|G) minus the probability of manifesting the disease if you do not have this specific genetic change P(D|G^c). This forms the numerator in formula 2c) below. In addition, a useful formula for penetrance should have output ranging from 0-100%. Therefore, a scaling factor is required for the denominator.

$$\text{Penetrance} = \frac{P(D|G)-P(D|G^c)}{1-P(D|G^c)} ,\qquad \text{(Formula 2c)}$$

Where the terms in the formula are defined in the main text of this article and at the end of Supplemental Methods 1.2.

At least two previous studies (8, 12) used this numerator, P(D|G) – P(D|G^c), to estimate penetrance, but the numerator does not represent a precise value for penetrance because the highest value of penetrance the numerator can achieve is less than 100%, due to the subtracted term P(D|G^C). The denominator is required as a scaling factor, which permits the formula to report penetrance estimates up to 100%. This scaling factor is an equivalent way of taking into account the possibility of dual (or multiple) diagnoses.

Formula 2a) can be shown to be equivalent to Formula 2c) using standard mathematical software, or by hand.

Supplemental Methods 1.4 – Similarity of this new penetrance formula to the attributable fraction among the exposed and risk difference

The concept of penetrance being the additional chance of a risk factor to cause an effect is not new. Two similar concepts, the attributable fraction among the exposed and the risk difference, have been well studied in contexts unrelated to genetic penetrance. Neither of these concepts exactly match that of genetic penetrance, so this section explores their similarities and differences.

The risk difference is the difference in risk of a condition between an exposed group and an unexposed group. Using the same notation as Supplemental Methods 1.2-1.3, the formula is:

$$Risk\ Difference = P(D|G) - \ P(D|G^c).$$

Compared to the penetrance Formula 2a) (Supplemental Methods 1.2), the risk difference does not have a weight. The weight term in the penetrance formula is needed because some genetic causes are 100% penetrant so subtraction of the P(D|G^c) term needs to be weighted to reflect this (Supplemental Methods 1.1-1.3). In contrast, statistical measures of risk difference do not take this into account. For example, if a poison kills everyone within 5 years, but 4% of controls will die from

other causes within 5 years, then penetrance for death for this poison will be 100%, whilst the risk difference is 96%.

The attributable fraction among the exposed is the proportion of incidents (people with the phenotype) in the exposed group (with the genetic change) that are attributable to the exposure (genetic change). Using the same notation as Supplemental Methods 1.2-1.3, the formula is:

$$Attributable\ fraction\ among\ the\ exposed = \frac{P(D|G) - P(D|G^c)}{P(D|G)}.$$

Compared to the penetrance Formula 2a) (Supplemental Methods 1.2), the attributable fraction among the exposed divides the numerator by its first term, to give the result as a fraction of the original. In contrast, the penetrance Formula 2a) does not divide the result in this manner. For example, if a poison kills 50% of the people who ingest it within 5 years, but 10% of controls will die from other causes within 5 years, then penetrance for death for this poison will be 0.5 - (1-0.5)/(1-0.1)*(0.1) = 44%, whilst the attributable fraction among the exposed is (0.5-0.1)/0.5 = 80%.

In summary, genetic penetrance is similar to other concepts in health and statistics. However, unique considerations in clinical genetics results in a different formula for penetrance compared to similar concepts in other fields. In genetics, penetrance ought to refer to the probability of a phenotype occurring due to the genetic change. When multiple genetic and environmental factors can cause the same phenotype, it can be useful to take into account whether the genetic change on its own might be able to cause the phenotype. These subtleties lead to the formulation of a penetrance formula that is distinct from similar mathematical or statistical formulas that are used in other fields of medicine.

Supplemental Methods 1.5 – Comparison of earlier concepts and formula for penetrance to the new concept and formula

Consider, as an example, the following two interpretations of penetrance if the phenotype is intellectual disability and if the discussion is about monogenic causes in a single gene.

Example 1) The penetrance of intellectual disability in this gene is the probability of manifesting the phenotype of intellectual disability <u>if you have</u> a pathogenic variant in this gene.

Example 2) The penetrance of intellectual disability in this gene is the probability of manifesting the phenotype of intellectual disability <u>due to</u> the pathogenic variant in this gene.

Both examples are compatible with the commonly published (ambiguous) definition of penetrance. However, Example 1 refers to the probability of having an intellectual disability, even if the reason for the intellectual disability was unrelated to the pathogenic variant. Example 2 would not include such an individual.

For example, an individual with a 15q11.2 duplication who happens to have an *SCN2A* monogenic cause for their epileptic encephalopathy will not be considered as being penetrant for the 15q11.2 duplication using this new definition (but was considered penetrant with the previous definition).

A slightly different example to demonstrate the difference would be an individual with a 15q11.2 duplication who happened to have an environmental cause for their intellectual disability (e.g. hypoxic ischemic brain injury at birth). Using the earlier definition of penetrance, this individual would be considered as penetrant for the 15q11.2 duplication. This is not the case with the new definition.

One issue that previous studies (19, 28, 33) have struggled with is whether to include individuals with (for example) a 15q11.2 duplication and an *NF1* deletion in datasets of penetrance for the 15q11.2 duplication. On the one hand, an individual with this dual diagnosis is clearly affected due to their *NF1* deletion (which causes Neurofibromatosis Type 1) – the 15q11.2 duplication probably plays a lesser or no role in this individual's symptoms. On the other hand, it is possible that these two genetic variants could interact in a way that results in a more severe phenotype. The earlier formula for penetrance would result in such individuals with dual pathology raising the penetrance estimate for the 15q11.2 duplication. The new formula allows inclusion of such individuals, but scales their relative contributions based on how penetrant the CNV (15q11.2 duplication) is for others in the cohort. For low-penetrant CNVs, the weight or the scaling factor acts in a manner that considers such individuals to have pathology largely due to their second cause and for highly-penetrant CNVs, the reverse is true. Therefore, individuals with multiple pathologies, whether these are environmental, monogenic or due to another CNV, can be included in datasets without altering the accuracy of penetrance calculations using this new formula. Therefore, this new formula provides increased utility compared to the earlier formula, and is accurate even when such individuals are included in the dataset for the affected cohort.

Supplemental Methods 1.6 – Negative penetrance estimates

Negative penetrance estimates occur when the genetic change protects against the phenotype; equivalently, when $P(D|G) < P(D|G^c)$.

Formula 2 is incorrect for negative values of penetrance. However, we show that in the most extreme clinical examples, the error remains less than 1%, which is small and therefore unlikely to be clinically relevant. Potential modifications to Formula 2 which may help to correct this error are suggested.

The maximal error occurs when $P(D|G)$ is 0%. That is, the maximal error occurs if the probability of disease when the genetic change is present equals 0%. Equivalently, the maximal error occurs when the genetic change is fully protective against the phenotype.

For ID, the probability of disease if the genetic change is not present, $P(D|G^c)$, is about 1.1%. Therefore formula 2 is,

$$Penetrance = P(D|G) - \frac{1 - P(D|G)}{1 - P(D|G^c)}.P(D|G^c)$$

$$Penetrance = 0 - \frac{1 - 0}{1 - 0.011}.(0.011)$$

$$Penetrance = -0.011122 = -1.1122\%$$

But this should be -0.011 (or -1.1%). The difference, in this example, is -0.000122, which is 0.0122% in absolute terms. Displayed in Table 1, the clinical difference, in absolute terms, is far less than 1% and unnoticeable. This is the maximal difference – it is unlikely that a genetic change will ever be fully protective against the phenotype – so any other value used to calculate this formula that results in negative penetrance estimates will be less extreme.

For conditions that are more prevalent than ID, a similar calculation can be undertaken. For all genetic scenarios where prevalence of disease is unlikely to be much higher, the difference will be <1% in absolute terms. That is, the negative penetrance estimate, if published in a manner similar to Table 1, will not report a figure that is more than 1% different from the true value.

Possible solutions to this incorrect formula for negative values of penetrance include using $P(D|G^C) - P(D|G)$ on the numerator instead, and/or $1-P(D|G)$ on the denominator instead. These may not be ideal solutions, so this area could be the focus of further research.

Supplemental Methods 2 – Studies used to estimate penetrance for CNVs associated with neurodevelopment

A systematic review (21) identified 9 studies that published data and penetrance estimates for CNVs associated with neurodevelopment. The present study used data from 8/9 of these (3, 4, 7, 10, 14, 15, 17, 19) to estimate penetrance for ID, excluding 1/9 studies (8) due to that study comprising individuals who had, "on average… moderate to severe autistic symptoms with relatively little intellectual disability."

In 1/8 of these included studies (19), the original dataset (35) reports only 423 individuals out of 90,172 with ID. Therefore, this subset of 423 individuals was extracted for analysis and the remainder discarded. In the remaining 7/8 studies (3, 4, 7, 10, 14, 15, 17), it was not possible to isolate a subset of individuals with intellectual disability from the affected cohorts. Since most individuals in these 7/8 studies were thought to have ID (Supplemental Methods 3.2), all participants from these 7/8 studies were included in the analysis. In total, affected cohort data from 8/9 earlier studies (3, 4, 7, 10, 14, 15, 17, 19) were compared against gnomAD v4.0 CNV controls (21, 33) to estimate penetrance in the present study. A more in-depth discussion of significant subsets of affected and control cohort data is presented below.

Datasets from Cooper et al. 2011 (3), Kirov et al. 2014 (4) and Rosenfeld et al. 2013(14):

It should be noted that the data from these studies all derive from Signature Laboratories, Spokane USA, and are likely to partially overlap. However, the CNV breakpoints in their studies sometimes differ and the three studies did not report on the same set of CNVs, which means each study contributed overlapping results from overlapping data sources. Collectively, these three studies report on penetrance estimates for 78 CNVs. Data from these three studies cannot be combined. A recent systematic review (21) chose to use data from Cooper et al. 2011 for 13/78 CNVs, Kirov et al. 2014 for 54/78 CNVs and Rosenfeld et al. 2013 for 11/78 CNVs. Our choice for which study's data to use for which CNV mirrored that of the systematic review (21).

Dataset from Martin et al. 2020 (19):

The study by Martin et al. 2020 (19) involved adults attending outpatient clinics serviced by one genetic laboratory in Pennsylvania USA. Sample size was unclear but involved more than 250,000 patients who were offered a chance to enrol in a genetic study on neuropsychiatric disorders, of whom 92,455 chose to accept. Of these, only individuals with clinical information and genomic data that passed quality filters were included. The net result was a cohort of 90,595 individuals that were presumed by the study authors to be representative of the general population. However, a high 13% (12,039/90,595) (19, 21) of individuals in this cohort were considered to be affected (this is high compared to other studies on CNV penetrance). These individuals had a documented neuropsychiatric issue in their medical record as defined by the DSM-V, which included ID, communication disorder, autism, attention deficit and hyperactivity disorder, specific language disorder, motor disorder, other neurodevelopmental disorder, schizophrenia, bipolar disorder, obsessive compulsive disorder, epilepsy and cerebral palsy. Restricting this to those with ID dropped this prevalence to 0.5% (423/90,595). This is lower than most estimates of ID prevalence. One possible reason might be that the lengthy consent process and focus group participation as a requirement of entry into the study, may have selected against those with ID, especially since most

of the >250,000 patients approached did not enrol in the study. Of the individuals in their cohort study who had ID, 58/423 harboured one of the 25 published CNVs in the study. It should be noted that the original 90,595 individuals forming the cohort by Martin et al. 2020 is very different from those of other studies (3, 4, 7, 10, 14, 15, 17), which were conducted as case series. In the former (19), few would have been candidates for genetic testing in the absence of consent for the study, whilst in studies conducted as a case series (3, 4, 7, 10, 14, 15, 17), all affected individuals received genetic testing as part of their enrolment in the study. However, by selecting those with ID in the cohort study by Martin et al. 2020 (19), the demographic in this cohort study becomes more similar to those in case studies (3, 4, 7, 10, 14, 15, 17) (as individuals with ID often meet criteria for genetic testing) and both types of studies can be pooled together for analysis.

Excluded affected cohort datasets:

It is noted that there are 5 studies (9, 11-13, 16) with published neurodevelopmental penetrance estimates for CNVs that were not included in the systematic review (21) due to irreproducibility of their penetrance estimates from their data or extreme bias. After consideration, these were also excluded from the current study.

gnomAD control dataset:

The gnomAD v4.0 CNV control cohort from an earlier systematic review (21, 33) was used in this study. Only the CNVs labelled by gnomAD as being from controls and with a CNV size < 10Mb are used (n=269,885), mirroring the approach used in the systematic review. Note that this dataset of n=269,885 individuals is a subset of the larger gnomAD dataset of n=464,297 individuals that is available online in the gnomAD browser (36) who have not been marked as controls. The gnomAD subset used in this study is therefore likely to contain fewer affected individuals than the larger dataset in the online gnomAD browser.

Genomic coordinates of 83 recurrent CNVs:

Genomic coordinates for CNVs sometimes differed between studies and (21)their original coordinates are listed in Supplemental Table 1 below.

Supplemental Table 1: Studies and genomic coordinates used for each CNV			
**Copy number variant**	**Study contributing data for the deletion,$^{d-}$ duplication^{d+} or both$^{d-/d+}$**	**Genomic coordinates (GRCh38) Mb**	**Size (Mb)**
1p36 deletion and 1p36 duplication [*GABRD*]	Kirov $^{d-/d+}$	1: 0.00-10.01	10
	gnomAD $^{d-/d+}$	c	c
1q21.1 proximal deletion and 1q21.1 proximal duplication [*RBM8A*]	Cooper $^{d-}$	(NCBI36) 1:144.0-144.3	c
	Rosenfeld $^{d+}$	(NCBI36) 1:144.0-144.5	c
	gnomAD $^{d-/d+}$	1:145.7-146.0	0.33
1q21.1 distal deletion and 1q21.1 distal duplication [*GJA5*]	Cooper $^{d-/d+}$	1:147.10-147.92	0.82
	Martin $^{d-/d+}$	1:147.11-147.92	0.81
	gnomAD $^{d-/d+}$	1:147.12-147.84	0.73
2p16.3 deletion [*NRXN1*]	Kirov $^{d-}$	2: 49.91-51.03	c
	gnomAD $^{d-}$	c	c
2q11.2 deletion [*TMEM127*]	Cooper $^{d-}$	2: 96.06-97.01	0.95
	gnomAD $^{d-}$	2: 96.11-96.99	0.87
2q13 proximal deletion and 2q13 proximal duplication [*NPHP1*]	Cooper $^{d-/d+}$	2:110.07-110.23	0.16
	gnomAD $^{d-/d+}$	2:110.12-110.21	0.08
2q23.1 deletion [*MBD5*]	Kirov $^{d-}$	2:148.0-148.5	0.55
	gnomAD $^{d-}$	c	c
2q37 deletion and 2q37 duplication [*HDAC4*]	Kirov $^{d-/d+}$	2:238.8-241.5	2.8
	gnomAD $^{d-/d+}$	2:239.1-241.9	2.8
3q29 deletion and 3q29 duplication [*DLG1*]	Kirov $^{d-/d+}$	3:196.00-197.61	1.6
	Martin $^{d-}$	3:196.03-197.62	1.6
	gnomAD $^{d-/d+}$	3:196.05-197.55	1.5
4p16.3 deletion (Wolf-Hirschhorn Syndrome) and 4p16.3 duplication	Kirov $^{d-/d+}$	4: 1.53-2.03	0.5
	gnomAD $^{d-/d+}$	4: 1.80-2.30	0.33
5q35.3 deletion (Sotos Syndrome) and 5q35.3 duplication [*NSD1*]	Kirov $^{d-/d+}$	5:176.29-177.63	1.4
	gnomAD $^{d-/d+}$	5:176.31-178.00	1.7
6p25 deletion and 6p25 duplication	Kirov $^{d-/d+}$	6: 0.16-6.06	5.9
	gnomAD $^{d-/d+}$	N/A	N/A
6q16 deletion and 6q16 duplication [*SIM1*]	Kirov $^{d-/d+}$	6:100.39-100.46	0.07
	gnomAD $^{d-/d+}$	c	c

7q11.23 deletion (Williams-Beuren Syndrome) and 7q11.23 duplication	Kirov [d-/d+]	7: 73.33-74.73	1.4
	Martin [d-/d+]	7: 73.33-74.73	1.4
	gnomAD [d-/d+]	7: 73.33-74.72	1.4
8p23.1 deletion and 8p23.1 duplication [*CLDN23*, *SOX7*, *GATA4*]	Kirov [d-/d+]	8: 8.23-12.03	3.8
	gnomAD [d-/d+]	c	c
9q34 deletion (Kleefstra syndrome) and duplication [*EHMT1*]	Kirov [d-/d+]	9:134.92-138.19	3.3
	gnomAD [d-/d+]	c	c
10q23 deletion and 10q23 duplication [*NRG3*, *GRID1*, *BMPR1A*]	Kirov [d-/d+]	10: 80.20-87.04	6.8
	Martin [d-]	10: 79.92-86.98	7.1
	gnomAD [d-/d+]	10: 79.94 - 87.09	7.2
13q12 deletion [*CRYL1*]	Cooper [d-]	13: 20.24-20.44	0.2
	gnomAD [d-]	c	c
15q11.2 deletion [BP1-BP2] and 15q11.2 duplication [*NIPA1*, *NIPA2*]	Cooper [d+]	15: 22.78-23.07	0.29
	Rosenfeld [d-]	15: 22.62-23.12	0.5
	Jonch [d-/d+]	15: 22.87-23.07	0.2
	gnomAD [d-/d+]	15: 22.79-23.04	0.25
15q11.2 deletion [BP1-BP3] and 15q11.2 duplication [*NIPA1*, *NIPA2*] (Prader-Willi Syndrome/Angelman Syndrome)	Isles [d+]	Unclear	Unclear
	Martin [d-/d+]	15: 22.78-28.14	5.4
	gnomAD [d-/d+]	15: 22.81-28.28	5.5
15q11q13 deletion [BP2-BP3] (Prader-Willi Syndrome/Angelman Syndrome) and 15q11.13 duplication	Kirov [d-/d+]	15: 24.57-28.18	3.6
	gnomAD [d-/d+]	c	c
15q13.3 deletion [BP4-BP5] and 15q13.3 duplication [*CHRNA7*]	Kirov [d-/d+]	15: 30.84-32.19	1.35
	Martin [d-]	15: 30.84-32.15	1.31
	gnomAD [d-/d+]	15: 30.63-32.11	1.49
15q13.3 smaller deletion and 15q13.3 smaller duplication [*CHRNA7* and *OTUD7A* only]	Kirov [d-/d+]	15: 31.72-32.16	0.44
	gnomAD [d-/d+]	c	c
15q24 deletion and 15q24 duplication [*BBS4*, *PML*, *SIN3A*]	Kirov [d-/d+]	15: 72.62-74.12	1.5
	Martin [d-]	15: 72.67-75.68	3.01
	gnomAD [d-/d+]	15: 72.69-75.21	2.53
15q24.2q24.5 deletion and 15q24.2q24.5 duplication [*FBXO22*, *TSPAN3*]	Kirov [d-/d+]	15: 75.68-77.91	2.23
	gnomAD [d-/d+]	15: 75.84-77.63	1.79

15q25.2 proximal deletion and 15q25.2 proximal duplication [*RPS17*, *HOMER2*, *BNC1*]	Kirov [d-/d+]	15: 82.51-84.07	1.56
	gnomAD [d-/d+]	15: 82.54-84.04	1.5
16p13.3 deletion (Rubinstein-Taybi Syndrome) [*CREBBP*]	Kirov [d-]	16: 3.73-3.88	0.15
	gnomAD [D-]	16: 3.73-3.81	0.08
16p13.11 deletion and 16p13.11 duplication [*MYH11*]	Rosenfeld [d-]	16: 14.90-16.40	1.5
	Kirov [d+]	16: 15.42-16.21	0.79
	Allach El Khattabi [d+]	c	0.16-2.62
	Martin [d-]	16: 15.42-16.20	0.78
	gnomAD [d-/d+]	16: 15.03-16.20	1.17
16p12.2 deletion (previously 16p12.1 deletion) and 16p12.2 duplication (previously 16p12.1 duplication) [*CDR2*]	Rosenfeld [d-]	16: 21.93-22.48	0.55
	Kirov [d+]	16: 21.93-22.45	0.52
	gnomAD [d-/d+]	16: 21.95-22.37	0.42
16p11.2p12.2 deletion (previously 16p11.2p12.1) and 16p11.2p12.2 duplication (previously 16p11.2p12.1 duplication)[a]	Kirov [d-/d+]	16: 21.52-29.09	7.57
	gnomAD [d-/d+]	c	c
16p11.2 distal deletion and 16p11.2 distal duplication [*SH2B1*]	Rosenfeld [d-/d+]	16: 28.73-29.08	0.35
	Martin [d-]	16: 28.81-29.04	0.23
	gnomAD [d-/d+]	16: 28.81-28.99	0.18
16p11.2 proximal deletion and 16p11.2 proximal duplication [*TBX6*]	Rosenfeld [d-/d+]	16: 29.58-30.23	0.65
	Martin [d-/d+]	16: 29.64-30.19	0.55
	gnomAD [d-/d+]	16: 29.66-30.19	0.52
17p13.3 deletion and 17p13.3 duplication [*YWHAE*]	Cooper [d+]	17: 0.65-1.45	0.8
	Kirov [d-]	17: 1.35-1.40	0.05
	gnomAD [d-/d+]	c	c
17p13.3 deletion and 17p13.3 duplication [*PAFAH1B1*]	Cooper [d+]	17: 2.46-3.02	0.56
	Kirov [d-]	17: 2.59-2.69	0.1
	gnomAD [d-/d+]	c	c
17p12 deletion (Hereditary Neuropathy with Liability to Pressure Palsies) and 17p12 duplication (Charcot Marie Tooth) [*PMP22*]	Cooper [d-/d+]	17: 14.17-15.60	1.43
	Martin [d+]	17: 14.19-15.52	1.33
	gnomAD [d-/d+]	17: 14.21-15.51	1.3

17p11.2 deletion (Smith-Magenis Syndrome) and 17p11.2 duplication (Potocki-Lupski Syndrome) [*RAI1*]	Kirov [d-/d+]	17: 16.92-18.38	1.46
	Martin [d-]	17: 16.91-20.30	3.39
	gnomAD [d-/d+]	17: 16.93-20.31	3.38
17q11.2 deletion (Neurofibromatosis Type 1) and 17q11.2 duplication [*NF1*]	Kirov [d-/d+]	17: 30.77-31.95	1.18
	Martin [d-/d+]	17: 30.78-31.94	1.16
	gnomAD [d-/d+]	17: 30.78-32.01	1.22
17q12 deletion (Renal Cysts and Diabetes) and 17q12 duplication [*HNF1B*]	Rosenfeld [d-/d+]	17: 36.36-37.87	1.51
	Martin [d-/d+]	17: 36.46-37.85	1.39
	gnomAD [d-/d+]	17: 36.49-37.75	1.26
17q21.31 deletion (Koolen-de Vries Syndrome) and 17q21.31 duplication [*MAPT*, *KANSL1*]	Kirov [d-/d+]	17: 45.62-46.10	0.48
	gnomAD [d-/d+]	c	c
17q23 deletion and 17q23 duplication [*TBX2*, *TBX4*]	Kirov [d-/d+]	17: 60.17-62.21	2.04
	gnomAD [d-/d+]	17: 60.04-62.14	2.1
19p13.12 deletion	Kirov [d-]	19: 12.97-16.59	3.62
	gnomAD [d-]	c	c
22q11.2 deletion Velocardiofacial Syndrome and 22q11.2 duplication [*TBX1*]	Rosenfeld [d+]	22: 18.34-21.22	2.88
	Kirov [d-]	22: 19.03-20.27	1.24
	Martin [d-/d+]	Unclear	Unclear
	gnomAD [d-/d+]	c	c
22q11.2 distal deletion and 22q11.2 distal duplication [*BCR*, *MAPK1*]	Kirov [d-/d+]	22: 21.56-23.31	1.75
	Martin [d-/d+]	22: 21.44-23.31	1.87
	gnomAD [d-/d+]	22: 21.56-23.38	1.82
22q13.33 deletion Phelan-McDermid Syndrome and 22q13.33 duplication [*SHANK3*]	Cooper [d-/d+]	22: 50.67-50.73	0.06
	gnomAD [d-/d+]	c	c
Xp22.3 duplication [*SHOX*]	Tropeano [d+]	X: 0.62-0.66	0.04-1.3

Supplemental Table 1: Table adapted from a systematic review (21). The table lists 83 CNVs, along with the genomic coordinates from various studies (3, 4, 7, 10, 14, 15, 17, 19) that supplied data for these CNVs. Where CNV sizes differ, the rationale for combining these as one CNV are discussed in the Supplemental Analysis in the systematic review (21).

c*: There are complexities in the genomic coordinates for this CNV, which are discussed in the Supplemental Analysis of a recent systematic review (21).*

[d-/d+]*: Study contributes data for the deletion (d-), duplication (d+) or both (d-/d+).*

NCBI36*: Coordinates provided by original study in NCBI36/hg18, which does not liftover appropriately to hg38.*

Supplemental Methods 3 – An exploration of the terms P(D) and P(G|D) used in the formula

Supplemental Methods 3.1-3.4 explores the terms in the formula and justifies the approach taken in this study in reporting penetrance estimates for recurrent CNVs. The aim is to provide a reason why we believe the prevalence of ID, at 1.1%, represents a more accurate choice for the prevalence of disease, P(D). This choice of 1.1% is one of two main changes responsible for updated penetrance estimates for recurrent CNVs.

Supplemental Methods 3.1 – Importance for the penetrance formula to refer to a single phenotype

The penetrance formula (both the earlier Formula 1 and newly proposed Formula 2) are most accurate when the data used to calculate penetrance represents a homogenous sample from a cohort of affected individuals. This can be seen from the mathematics used in the construction of the formula. Formula 1 (the earlier formula used by previous studies) is presented below for convenience:

Penetrance = $\frac{P(G|D).P(D)}{P(G)}$. Formula 1 (earlier formula for penetrance)

The symbol P refers to probability, G refers to Genotype (or CNV) and D refers to Disease (or phenotype). The terms are explained in the main text of this study and Supplemental Methods 1.2.

A homogenous sampling of the affected cohort is simpler if only a single phenotype needs to be considered. If the formula is being used to calculate penetrance of multiple phenotypes, then the formula is accurate only if the affected cohort is sampled in a manner representative of the respective frequencies of these phenotypes in the general population. For example, if the ratio of individuals who have 'ID and cardiac defects', 'ID without cardiac defects' and 'cardiac defects without ID' is 1:3:2, then the data for the affected cohort P(G|D) used in the formula should be pooled from a group that has a similar frequency of these subsets. Adding more phenotypes makes this more complex, as the homogeneity has to hold across all combinations of all phenotypes. In addition, if the same data is being used to calculate penetrance for multiple CNVs, then these phenotypes have to be present in the same proportion for all CNVs, which is impossible.

As a concrete (exaggerated) example, consider an affected cohort P(G|D) of 10,000 people, comprised of 9,995 with ID, 3 with autism (but not ID) and 2 with congenital malformations (but not ID nor autism). The prevalence of disease, P(D), should take a value of 1.1% (as this is a reasonable estimate of the prevalence of ID), but the inclusion of a few individuals with other phenotypes means the choice of P(D) = 4% or 5% might be chosen in an attempt to include these additional phenotypes (3, 4, 7, 10, 11, 14, 15, 17). For low penetrant CNVs, using 5% rather than 1.1% in Formula 1 leads to a 5-fold increase in penetrance estimates. This is clearly an inflated result from the addition of so few individuals without ID to an affected cohort composed primarily of ID. *For a choice of P(D) = 4% or 5% to hold, there needs to be 4-5 times more individuals in the affected cohort without ID than with ID.*

This example demonstrates that the affected cohort has to be homogenous in the phenotype(s) D that the formula is estimating the penetrance of – if D is a combination of multiple phenotypes, then the affected cohort has to be sampled from a population that represents the relative frequencies of those phenotypes. Interestingly, this point was raised in a letter to the editor in 2013 (24) regarding a study on CNV penetrance (14), though this is the first time it has been described in detail since.

Therefore, penetrance for multiple phenotypes are difficult to estimate using this formula. The penetrance formulas (both the earlier formula and the newly proposed formula) are most accurate when calculating penetrance of a single phenotype.

Supplemental Methods 3.2 – Composition of affected cohorts

Of the 8 studies contributing data for affected cohorts, 3 studies contribute most of the data (3, 4, 14) (Supplemental Table 2).

- All 3/8 of the main studies (3, 4, 14) are primarily comprised of individuals who have ID or developmental delay. In one of the studies (3), the percentages of each phenotype add exactly to 100%, meaning individuals with more than one phenotype were included in one category only, thereby raising the possibility that there may be more than the listed percentage who had ID. In another study (14), ID forms the main category, with epilepsy, autism, congenital anomalies, dysmorphic features or unspecified being the other categories. During the study period of 2004-2012, many neonates would have received chromosomal microarray for these indications and may later develop ID. Older children (or adults) may also have had ID but not have this listed on their pathology request form. Therefore, the study (14) noted that the listed percentages of ID and developmental delay was likely higher than indicated.
- For the Martin et al. 2020 study (19), only the ID subset of their affected cohort was used as data in the present study. Therefore, all individuals had ID.
- For the Jonch et al. 2019 study (17), their affected cohort data consisted of the SJCHU and OUH cohorts, all of whom had ID or developmental delay.
- For the Isles et al. 2016 study (10), their affected cohort data consisted of pooled affected cohort data from 2 other studies (37, 38), with the phenotype subset of the UK BBGRE study (38) at the time when data was collected unable to be determined.
- For the Allach El Khattabi et al. 2018 study (7), all had ID, developmental delay or autism. Although many individuals with autism today do not have ID, those who were recruited by geneticists during the era when microarray technology was new, were more likely to have concurrent ID or more severe autism. So this cohort was likely to be primarily a cohort with ID, developmental delay, or autism with concurrent ID.

Supplemental Table 2: Composition of affected cohorts that contribute data for this publication

**Study first author & year**	**Laboratory**	**Time period**	**Total number in affected cohort**	**Number of CNVs that the study contributes**	**Composition of affected cohort**	
			15767	13[S]	ID, autism, dev delay	73%

Cooper 2011 (corrigendum 2013) (3)	Signature Laboratories, Spokane USA	Unclear (pre-2011)			Congenital malformation, hypotonia, feeding difficulties, growth retardation, cardiovascular anomalies, renal anomalies, behavioural issues and other	15%
					Unspecified	12%
Kirov 2014 (4)	Signature Laboratories, Spokane USA	2008-2010	6623 – 32587[K]	54[S]	Developmental delay or autism (with or without congenital malformations)	100%
Rosenfeld 2013 (14)	Signature Laboratories, Spokane USA	2004-2012	48637	11[S]	Developmental delay or ID	51%, 54%[R]
					Epilepsy	10%, 11%[R]
					Autism 10-14%	10%, 14%[R]
					Congenital anomalies	16%, 23%[R]
					Dysmorphic features	25%, 16%[R]
					Unspecified	7%, 5%[R]
Martin 2020 (19)	Geisinger Health System Laboratory, Danville USA	2007-2017	423[M]	25	ID	100%
Jonch 2019 (17)	Saint-Justine University Hospital and Odense University Hospital	Unclear	15448	2	Developmental delay or ID	100%
Isles 2016 (10)	Many laboratories globally (37, 38)	Unclear	50995	1	Developmental delay or ID	Unclear
					Unspecified	Unclear
Tropeano 2016 (15)	Guy's and St Thomas' National Health Service Foundation Trust (London) and Hospital for Sick Children (Toronto)	Unclear	18857	1	Neurodevelopmental disability	100%

Allach El Khattabi 2018 (7)	Unclear	Unclear	16013	1	Developmental delay, ID or autism	100%

Supplemental Table 2: Composition of the affected cohort for the 8 studies that contribute to the present study. Note that the percentages listed in one study (3) add precisely to 100%, indicating that individuals with a dual diagnosis were included in one of these categories only, so the listed percentage may be an under-representation of the true percentage for that phenotype. It was also noted in one of the studies (14), that the percentages are likely to be underestimates of the true burden of disease due to lack of clinical information on the request forms. This observation likely extends to that of other studies in this table.

[K]: This study had different numbers of affected individuals, depending on the CNV reported.

[M]: The 423 individuals in this study with ID were extracted from their original publication (See Supplemental Methods 2).

[R]: This study did not list a breakdown of their total affected cohort's phenotype. Instead, they list two numbers that show the percentage of referrals for 3 months in 2008, and for 3 months in 2011, which may or may not be reflective of their whole cohort.

[S]: Note that data from 3 studies (3, 4, 14) are likely to partially overlap, as they come from Signature Laboratories, Spokane USA. For each CNV, only one of these studies, at most, is used as affected cohort data.

In summary, the majority of affected cohorts used in this study had ID or developmental delay. Of those that are not listed as having this, arguments have been outlined to highlight many reasons to suspect that some might have had ID or developmental delay regardless. Taken in aggregate, it is likely that a clear majority of the affected cohort had ID or developmental delay.

An in-depth consideration of alternate ways of managing the non-ID subset of these patients is discussed in Supplemental Discussion 1.2 and 1.3. The conclusion after considering other approaches is that the data is imperfect, so a perfect solution is impossible. However, if most of the affected cohort have ID, and if a single phenotype is needed to be chosen for the penetrance formula to be accurate, then this supplemental chapter demonstrates that the optimal single phenotype of choice is ID.

Supplemental Methods 3.3 – Prevalence of intellectual disability

The prevalence of intellectual disability, P(D), was estimated to be 1.1% in this study (Supplemental Table 3). This was based on the median value of the prevalence of ID in children in high income nations calculated from data in a systematic review (20). The choice to restrict this to studies of children in high income nations was because most of the affected cohort data used in the formula for penetrance involved children from high income nations. Therefore, for the terms P(D), P(G|D) and $P(G|D^c)$ in the formula to be consistent, they should refer to the same demographic.

Supplemental Table 3: Studies contributing to a systematic review of prevalence for pediatric intellectual disability in high income nations.

Authors	Country	Prevalence
Andersen, Fledelius, Fons, and Haugsted (1990)	Denmark	0.44%
Baird and Sadovnick (1985)	Canada	0.56%
Blomquist, Gustavson, and Holmgren (1981)	Sweden	0.76%
Bradley, Thompson, and Bryson (2002)	Canada	0.72%
Camp, Broman, Nichols, and Leff (1998)	USA	3.68%
Cooper (1990)	Germany	0.70%
Hagberg, Lewerth, Olsson, and Westerberg (1987)	Sweden	0.72%
Heikura et al. (2003)	Finland	1.11%
Hou, Wang, and Chuang (1998)	Taiwan	2.81%
Kaariainan (1987)	Finland	1.38%
Leonard, Petterson, Bower, and Sanders (2003)	Australia	1.43%
Murphy, Yeargin-Allsopp, Decoufle, and Drews (1995)	USA	1.20%
Petterson, Bourke, Leonard, Jacoby, and Bower (2007)	Australia	1.29%
Rantakallio and von Wendt (1985)	Finland	1.08%
Shiotsuki et al. (1984)	Japan	0.71%
Stromme and Valvatne (1998)	Norway	0.62%
Tomas Vila, Paricio Talayero, Colomer Revuelta, Andres Celma, and Moratal (1991)	Spain	1.36%
**Median value**		**1.08%**

In a systematic review (20) of the prevalence for intellectual disability, 17 studies of children in high income nations were identified. The median prevalence of ID was 1.08%, which was rounded to 1.1% for the value of P(D) used in this study.

Supplemental Methods 3.4 – Penetrance for autism and schizophrenia

Some studies publish separate CNV penetrance estimates for autism and/or schizophrenia (4, 5, 8, 10, 15, 19). CNV penetrance estimates for autism and schizophrenia are unlikely to be as accurate as CNV penetrance estimates for ID due to numerous reasons.

The diagnostic criteria for autism, has changed several times over the last few decades. One generation ago, the definition was such that individuals with autism rarely attained adult self-sufficiency or independence (39), with a prevalence of <1/1000 according to diagnostic criteria at the time (40). In contrast, most individuals with autism today do not have an intellectual disability (41, 42) and the prevalence is closer to 1/100 (43). Today, many individuals with autism are able to function independently as adults (41). It is likely that an expanded definition, increased recognition of the disorder and early intervention has led to milder forms of this condition being diagnosed in children and adults compared to earlier studies (44-46). This means studies on autism that mix cohorts of children and adults may be studying different conditions. Also, individuals with mild autism are rarely referred for chromosomal microarray in comparison to individuals with severe autism and comorbid intellectual disability. Taken in aggregate, this suggests genetic studies of

affected cohorts of individuals with autism may represent a non-heterogenous mix of autism – biased in number towards younger children with a more inclusive definition of autism for some studies, and simultaneously potentially biased towards those with more severe autism in other studies. In addition to unclear affected cohorts, control cohorts of adults without known autism based on earlier criteria, could actually include adults who may satisfy the definition of autism by today's standards. Therefore, penetrance estimates for autism using the Bayesian formula for penetrance faces additional challenges.

Schizophrenia is primarily an adult-onset condition. The use of the Bayesian formula for penetrance (both the earlier Formula 1 and the new Formula 2) relies on a control cohort that does not have schizophrenia. This can be challenging since young adults in a control cohort may develop schizophrenia later in life. A recent systematic review of CNV penetrance identified several CNVs that predisposed to adult onset conditions (e.g. 17p12 deletion [*PMP22*] that causes Hereditary Neuropathy with Liability to Pressure Palsies and the reciprocal duplication that causes Charcot Marie Tooth Disease) and noted that penetrance using this formula for these adult onset conditions were 2% and 11% respectively (21), which was clearly incorrect as these conditions are more highly penetrant (22, 23, 47). This was due to affected individuals in the control cohorts, who could have been pre-symptomatic or have subclinical symptoms. This example illustrates that the Bayesian formulation for penetrance is not suited to estimate penetrance of adult-onset conditions without modification.

Similar observations for autism and schizophrenia have been made previously in a letter to the editor regarding neurodevelopmental CNVs (24).

Supplemental Results 1 – Supplemental table of penetrance estimates with different values of P(D)

The Results section of the main paper includes Table 1, which lists 83 CNVs along with their corresponding penetrance estimates for ID. Some CNVs in Table 1 have censored results because they are either not associated with ID or are primarily linked to a non-ID phenotype. Supplemental Table 4 (below) presents penetrance estimates for all 83 CNVs using the new formula across a range of values for P(D), irrespective of phenotype. These penetrance estimates may be useful in niche cases (as discussed in Supplemental Discussion 1.2), but only if the affected cohort (i) accurately reflects the CNV's phenotype, (ii) does not contain (or only contains a few) individuals with phenotypes unrelated to the CNV, and (iii) the control cohort lacks individuals with the CNV phenotype. However, for most CNVs, the values provided in Table 1 of the Results section in the main paper are expected to be more applicable.

Supplemental Table 4: Penetrance estimates of 83 recurrent CNVs using the new formula and varying levels of prevalence of disease

Copy number variant	Size (Mb)	Deletion/ Duplication	Pooled affected cohort	gnomAD controls	Penetrance using new formula and P(D) = 1.1% (95% confidence interval)	Penetrance using new formula and P(D) = 2% (95% confidence interval)	Penetrance using new formula and P(D) = 3% (95% confidence interval)	Penetrance using new formula and P(D) = 4% (95% confidence interval)	Penetrance using new formula and P(D) = 5.08% (95% confidence interval)
1p36 deletion and 1p36 duplication [*GABRD*] [a]	10*	Del	78/ 32587	0/ 269885	100%* (76 - 100)	100%* (85 - 100)	100%* (90 - 100)	100%* (92 - 100)	100%* (94 - 100)
		Dup	16/ 15767	0/ 269885	100%* (52 - 100)	100%* (66 - 100)	100%* (75 - 100)	100%* (80 - 100)	100%* (83 - 100)
1q21.1 proximal deletion and 1q21.1 proximal duplication [*RBM8A*] [a]	*	Del	13/ 15767	53/ 269885	3.4%* (1.1 - 6.3)	6.0%* (1.9 - 11)	8.8%* (2.9 - 16)	11%* (3.8 - 20)	14%* (4.8 - 24)
		Dup	85/ 48637	249/ 269885	1.0%* (0.5 - 1.5)	1.8%* (0.9 - 2.7)	2.6%* (1.4 - 4.0)	3.5%* (1.8 - 5.3)	4.3%* (2.3 - 6.7)

1q21.1 distal deletion and 1q21.1 distal duplication [*GJA5]* [a]	0.82	Del	49/ 16190	71/ 269885	10% (7.1 - 15)	17% (12 - 24)	24% (17 - 32)	30% (22 - 39)	35% (26 - 44)
		Dup	28/ 16190	94/ 269885	4.2% (2.2 - 6.5)	7.4% (4.0 - 11)	11% (5.9 - 16)	14% (7.7 - 20)	17% (9.5 - 24)
2p16.3 deletion [*NRXN1]* [a]	*	Del	12/ 6623	45/ 269885	9.8%* (4.4 - 17)	16%* (7.6 - 27)	23%* (11 - 36)	28%* (14 - 43)	33%* (17 - 48)
2q11.2 deletion [*TMEM127*] [a]	0.95	Del	2/ 15767	16/ 269885	1.2% [NS] (-1.1 - 5.9)	2.2%[NS] (-2.0 - 10)	3.3%[NS] (-3.1 - 15)	4.4%[NS] (-4.2 - 19)	5.5%[NS] (-5.4 - 22)
2q13 proximal deletion and 2q13 proximal duplication [*NPHP1*] [a]	0.16	Del	78/ 15767	1533/ 269885	-0.1% [NS] (-0.4 - 0.1)	-0.3% (-0.6 - 0.2)	-0.4% (-1.0 - 0.2)	-0.5% (-1.3 - 0.3)	-0.7% (-1.7 - 0.4)
		Dup	118/ 15767	1763/ 269885	0.2% [NS] (-0.1 - 0.4)	0.3% [NS] (-0.1 - 0.7)	0.4% [NS] (-0.2 - 1.1)	0.6% [NS] (-0.2 - 1.5)	0.7% [NS] (-0.3 - 1.9)
2q23.1 deletion [*MBD5*]	0.55 *	Del	20/ 32587	0/ 269885	100% (42 - 100)	100% (57 - 100)	100% (66 - 100)	100% (72 - 100)	100% (77 - 100)
2q37 deletion and 2q37 duplication [*HDAC4*] [a]	2.8	Del	20/ 32587	0/ 269885	100%* (42 - 100)	100%* (57 - 100)	100%* (66 - 100)	100%* (72 - 100)	100%* (77 - 100)
		Dup	2/ 32587	1/ 269885	15%* [NS] (-1.1 - 100)	24%* [NS] (-2.0 - 100)	32%* [NS] (-3.1 - 100)	38%* [NS] (-4.2 - 100)	44%* [NS] (-5.4 - 100)
3q29 deletion and 3q29 duplication [*DLG1*] [a]	1.6	Del	20/ 33010	6/ 269885	22%* (11 - 52)	34%* (18 - 67)	44%* (25 - 75)	51%* (30 - 80)	57%* (36 - 84)
		Dup	18/ 32587	4/ 269885	29%* (13 - 67)	42%* (22 - 79)	52%* (29 - 85)	59%* (36 - 88)	65%* (41 - 90)
4p16.3 deletion (Wolf-Hirschhorn Syndrome) and 4p16.3 duplication [a]	0.5	Del	17/ 32587	0/ 269885	100% (38 - 100)	100% (52 - 100)	100% (62 - 100)	100% (69 - 100)	100% (74 - 100)
		Dup	4/ 32587	3/ 269885	9.9% (0.7 - 100)	17% (1.3 - 100)	23% (1.9 - 100)	29% (2.6 - 100)	34% (3.2 - 100)
5q35.3 deletion (Sotos Syndrome) and 5q35.3 duplication [*NSD1*] [a]	1.4 or 1.7*	Del	14/ 32587	0/ 269885	100% (31 - 100)	100% (45 - 100)	100% (55 - 100)	100% (62 - 100)	100% (67 - 100)
		Dup	4/ 32587	0/ 269885	100%* (7.4 - 100)	100%* (13 - 100)	100%* (18 - 100)	100%* (23 - 100)	100%* (27 - 100)

6p25 deletion and 6p25 duplication	5.9	Del	23/ 32587	0/ 269885	100% (46 - 100)	100% (61 - 100)	100% (70 - 100)	100% (76 - 100)	100% (80 - 100)
		Dup	12/ 32587	0/ 269885	100%* (29 - 100)	100%* (42 - 100)	100%* (52 - 100)	100%* (59 - 100)	100%* (65 - 100)
6q16 deletion and 6q16 duplication [*SIM1*] [a]	0.07 *	Del	1/ 23380	3/ 269885	3.0% [NS] (-1.1 - 100)	5.4% [NS] (-2.0 - 100)	7.9% [NS] (-3.1 - 100)	10% [NS] (-4.2 - 100)	13% [NS] (-5.4 - 100)
		Dup	1/ 23380	5/ 269885	1.4% [NS] (-1.1 - 10)	2.6% [NS] (-2.0 - 17)	3.8% [NS] (-3.1 - 24)	5.0% [NS] (-4.2 - 30)	6.2% [NS] (-5.4 - 35)
7q11.23 deletion (Williams-Beuren Syndrome) and 7q11.23 duplication [a]	1.4	Del	84/ 33010	2/ 269885	79%* (59 - 100)	87%* (72 - 100)	91%* (79 - 100)	93%* (84 - 100)	95%* (87 - 100)
		Dup	41/ 33010	7/ 269885	34%* (20 - 62)	48%* (31 - 75)	58%* (41 - 82)	65%* (48 - 85)	70%* (54 - 88)
8p23.1 deletion and 8p23.1 duplication [*CLDN23*, *SOX7*, *GATA4*] [a]	3.8	Del	18/ 32587	0/ 269885	100%* (39 - 100)	100%* (53 - 100)	100%* (63 - 100)	100%* (70 - 100)	100%* (74 - 100)
		Dup	24/ 32587	0/ 269885	100%* (47 - 100)	100%* (62 - 100)	100%* (71 - 100)	100%* (76 - 100)	100%* (80 - 100)
9q34 deletion (Kleefstra syndrome) and duplication [*EHMT1]* [a]	3.3	Del	18/ 32587	0/ 269885	100% (38 - 100)	100% (53 - 100)	100% (63 - 100)	100% (69 - 100)	100% (74 - 100)
		Dup	8/ 32587	0/ 269885	100%* (19 - 100)	100%* (31 - 100)	100%* (40 - 100)	100%* (47 - 100)	100%* (53 - 100)
10q23 deletion and 10q23 duplication [*NRG3*, *GRID1*, *BMPR1A*][a]	6.8 or 7.1 or 7.2*	Del	28/ 33010	2/ 269885	56%* (30 - 100)	69%* (44 - 100)	77%* (54 - 100)	82%* (61 - 100)	85%* (66 - 100)
		Dup	5/ 32587	6/ 269885	6.1%* (0.5 - 22)	11%* (0.9 - 34)	15%* (1.3 - 44)	19%* (1.7 - 51)	23%* (2.2 - 57)
13q12 deletion [*CRYL1*] [a]	0.2	Del	14/ 15767	227/ 269885	0.1% [NS] (-0.5 - 0.8)	0.1% [NS] (-0.9 - 1.4)	0.2% [NS] (-1.4 - 2.0)	0.2% [NS] (-1.9 - 2.7)	0.3% [NS] (-2.4 - 3.4)
15q11.2 deletion [BP1-BP2] and 15q11.2 duplication [*NIPA1*, *NIPA2*] [a]	0.2 or 0.25 or 0.29 or 0.5*	Del	312/ 40561	956/ 269885	1.3% (1.0 - 1.6)	2.3% (1.8 - 2.8)	3.4% (2.7 - 4.2)	4.5% (3.5 - 5.5)	5.6% (4.4 - 6.9)
		Dup	149/ 31215	1331/ 269885	0% [NS] (-0.2 - 0.1)	-0.1% [NS] (-0.4 - 0.3)	-0.1% [NS] (-0.6 - 0.4)	-0.1% [NS] (-0.8 - 0.5)	-0.2% [NS] (-1.0 - 0.7)

15q11.2 deletion [BP1-BP3] and 15q11.2 duplication [*NIPA1*, *NIPA2*] (Prader-Willi Syndrome/Angelman Syndrome) [a]	5.4 or 5.5*	Del	4/ 423	0/ 269885	100% (88 - 100)	100% (93 - 100)	100% (95 - 100)	100% (96 - 100)	100% (97 - 100)
		Dup	54/ 51418	11/ 269885	21%* (13 - 39)	33%* (22 - 54)	43%* (29 - 64)	50%* (35 - 70)	56%* (41 - 75)
15q11q13 deletion [BP2-BP3] (Prader-Willi Syndrome/Angelman Syndrome) and 15q11.13 duplication [a]	3.6	Del	60/ 32587	0/ 269885	100% (70 - 100)	100% (81 - 100)	100% (87 - 100)	100% (90 - 100)	100% (92 - 100)
		Dup	82/ 32587	11/ 269885	40%* (28 - 60)	55%* (41 - 73)	65%* (51 - 80)	71%* (58 - 84)	76%* (64 - 87)
15q13.3 deletion [BP4-BP5] and 15q13.3 duplication [*CHRNA7*] [a]	1.31 or 1.35 or 1.49 *	Del	87/ 33010	27/ 269885	22%* (15 - 31)	34%* (25 - 45)	43%* (33 - 55)	50%* (40 - 62)	56%* (46 - 68)
		Dup	27/ 32587	140/ 269885	0.7%* (0.0001 - 1.4)	1.2%* (0 - 2.6)	1.8%* (0 - 3.8)	2.3%* (0 - 5.0)	2.9%* (0 - 6.3)
15q13.3 smaller deletion and 15q13.3 smaller duplication [*CHRNA7* and *OTUD7A* only] [a]	0.44 *	Del	7/ 23380	0/ 269885	*	*	*	*	*
		Dup	*	*	*	*	*	*	*
15q24 deletion and 15q24 duplication [*BBS4*, *PML*, *SIN3A*] [a]	1.5 or 2.53 or 3.01 *	Del	10/ 33010	0/ 269885	100% (23 - 100)	100% (36 - 100)	100% (45 - 100)	100% (52 - 100)	100% (58 - 100)
		Dup	4/ 32587	3/ 269885	9.9%* (0.4 - 100)	17%* (0.8 - 100)	23%* (1.1 - 100)	29%* (1.5 - 100)	34%* (1.9 - 100)

15q24.2q24.5 deletion and 15q24.2q24.5 duplication [*FBXO22*, *TSPAN3*] [a]	1.79 or 2.23	Del	5/ 32587	1/ 269885	31%* (6.6 - 100)	45%* (11 - 100)	55%* (16 - 100)	62%* (21 - 100)	67%* (25 - 100)
		Dup	6/ 32587	1/ 269885	35%* (7.4 - 100)	49%* (13 - 100)	59%* (18 - 100)	66%* (23 - 100)	71%* (27 - 100)
15q25.2 proximal deletion and 15q25.2 proximal duplication [*RPS17*, *HOMER2*, *BNC1*] [a]	1.56	Del	2/ 23380	0/ 269885	100% [NS] (-1.1 - 100)	100% [NS] (-2.0 - 100)	100% [NS] (-3.1 - 100)	100% [NS] (-4.2 - 100)	100% [NS] (-5.4 - 100)
		Dup	4/ 23380	1/ 269885	33%* (5.0 - 100)	47%* (8.7 - 100)	58%* (13 - 100)	64%* (16 - 100)	70%* (20 - 100)
16p13.3 deletion (Rubinstein-Taybi Syndrome) [*CREBBP*] [a]	0.1	Del	10/ 32587	1/ 269885	47%* [b] (17 - 100)	62%* [b] (27 - 100)	71%* [b] (35 - 100)	77%* [b] (42 - 100)	81%* [b] (48 - 100)
16p13.11 deletion and 16p13.11 duplication [*MYH11*] [K]	0.16-2.62 *	Del	53/ 33649	83/ 269885	4.3%* (2.7 - 6.4)	7.6%* (4.9 - 11)	11%* (7.1 - 16)	14%* (9.3 - 20)	17%* (12 - 24)
		Dup	143/ 48600	398/ 269885	1.1%* (0.7 - 1.5)	2.0%* (1.3 - 2.7)	2.9%* (1.9 - 4.0)	3.8%* (2.5 - 5.2)	4.8%* (3.1 - 6.6)
16p12.2 deletion (previously 16p12.1 deletion) and 16p12.2 duplication (previously 16p12.1 duplication) [*CDR2*] [a]	0.42 or 0.52 or 0.55 *	Del	62/ 33226	139/ 269885	2.8%* (1.8 - 4.0)	5.0%* (3.2 - 7.1)	7.3%* (4.7 - 10)	9.5%* (6.1 - 13)	12%* (7.6 - 16)
		Dup	16/ 32587	135/ 269885	0% [NS] (-0.5 - 0.6)	0% [NS] (-1.0 - 1.1)	-0.1% [NS] (-1.5 - 1.6)	-0.1% [NS] (-2.0 - 2.1)	-0.1% [NS] (-2.5 - 2.7)

16p11.2p12.2 deletion (previously 16p11.2p12.1) and 16p11.2p12.2 duplication (previously 16p11.2p12.1 duplication) [a]	7.57	Del	20/ 32587	0/ 269885	100% (42 - 100)	100% (57 - 100)	100% (66 - 100)	100% (72 - 100)	100% (77 - 100)
		Dup	14/ 32587	0/ 269885	100% (33 - 100)	100% (47 - 100)	100% (57 - 100)	100% (64 - 100)	100% (69 - 100)
16p11.2 distal deletion and 16p11.2 distal duplication [*SH2B1*] [a]	0.23 *	Del	46/ 33649	32/ 269885	10%* (6.6 - 16)	17%* (11 - 26)	24%* (16 - 35)	30%* (20 - 41)	35%* (25 - 47)
		Dup	35/ 33226	81/ 269885	2.7%* (1.4 - 4.4)	4.8%* (2.5 - 7.7)	7.0%* (3.7 - 11)	9.1%* (4.9 - 14)	11%* (6.1 - 17)
16p11.2 proximal deletion and 16p11.2 proximal duplication [*TBX6*] [a]	0.55 or 0.65 *	Del	155/ 33649	58/ 269885	18%* (14 - 24)	29%* (23 - 36)	38%* (31 - 46)	45%* (38 - 53)	51%* (43 - 59)
		Dup	95/ 33649	73/ 269885	9.4% (6.9 - 13)	16% (12 - 21)	22% (17 - 28)	27% (21 - 35)	32% (25 - 40)
17p13.3 deletion and 17p13.3 duplication [*YWHAE*][a]	0.05 or 0.8*	Del	7/ 32587	0/ 269885	100% (15 - 100)	100% (24 - 100)	100% (32 - 100)	100% (38 - 100)	100% (44 - 100)
		Dup	6/ 15767	8/ 269885	12%* (2.5 - 31)	19%* (4.5 - 45)	26%* (6.6 - 55)	32%* (8.7 - 62)	38%* (11 - 67)
17p13.3 deletion and 17p13.3 duplication [*PAFAH1B1*]	0.1 or 0.56 *	Del	8/ 32587	0/ 269885	100% (18 - 100)	100% (28 - 100)	100% (37 - 100)	100% (44 - 100)	100% (50 - 100)
		Dup	4/ 15767	1/ 269885	43%* (7.7 - 100)	57% (13 - 100)	67% (18 - 100)	73% (23 - 100)	77% (28 - 100)
17p12 deletion (Hereditary Neuropathy with Liability to Pressure Palsies) and 17p12 duplication (Charcot Marie Tooth) [*PMP22*] [a]	1.33 or 1.43	Del	3/ 15767	146/ 269885	-0.7% [NS] (-1.1 to -0.2)	-1.3% [NS] (-2.0 to -0.4)	-2.0% [NS] (-3.1 to -0.6)	-2.7% [NS] (-4.2 to -0.8)	-3.4% [NS] (-5.4 to -1.0)
		Dup	9/ 16190	75/ 269885	1.1% [NS] (-0.2 - 2.8)	2.0% [NS] (-0.3 - 4.9)	2.9% [NS] (-0.5 - 7.2)	3.8% [NS] (-0.7 - 9.3)	4.8% [NS] (-0.9 - 12)

17p11.2 deletion (Smith-Magenis Syndrome) and 17p11.2 duplication (Potocki-Lupski Syndrome) [*RAI1*] [a]	1.46 or 3.39 *	Del	35/ 33010	0/ 269885	100% (56 - 100)	100% (70 - 100)	100% (78 - 100)	100% (82 - 100)	100% (85 - 100)
		Dup	25/ 32587	1/ 269885	69%* (38 - 100)	80%* (52 - 100)	86%* (62 - 100)	89%* (69 - 100)	91%* (74 - 100)
17q11.2 deletion (Neurofibromatosis Type 1) and 17q11.2 duplication [*NF1*] [a]	1.2	Del	26/ 33010	2/ 269885	54%* (28 - 100)	68%* (41 - 100)	76%* (51 - 100)	81%* (58 - 100)	84%* (64 - 100)
		Dup	35/ 33010	4/ 269885	44%* (25 - 79)	59%* (38 - 88)	68%* (48 - 91)	74%* (55 - 93)	78%* (61 - 95)
17q12 deletion (Renal Cysts and Diabetes) and 17q12 duplication [*HNF1B*][a]	1.26 or 1.39 or 1.51	Del	29/ 33649	6/ 269885	29%* (16 - 59)	43%* (26 - 73)	53%* (34 - 80)	60%* (41 - 84)	66%* (47 - 87)
		Dup	38/ 33649	61/ 269885	4.2% (2.4 - 6.6)	7.4% (4.3 - 11)	11% (6.3 - 16)	14% (8.3 - 20)	17% (10 - 25)
17q21.31 deletion (Koolen-de Vries Syndrome) and 17q21.31 duplication [*MAPT*, *KANSL1*] [a]	0.17 or 0.48 *	Del	42/ 32587	0/ 269885	100% (62 - 100)	100% (75 - 100)	100% (82 - 100)	100% (85 - 100)	100% (88 - 100)
		Dup	5/ 32587	1/ 269885	31% (7.0 - 100)	45% (12 - 100)	55% (17 - 100)	62% (22 - 100)	67% (26 - 100)
17q23 deletion and 17q23 duplication [*TBX2*, *TBX4*]	2.04 or 2.1	Del	6/ 32587	0/ 269885	100% (15 - 100)	100% (24 - 100)	100% (32 - 100)	100% (38 - 100)	100% (44 - 100)
		Dup	1/ 32587	1/ 269885	7.4%* [NS] (-1.1 - 100)	13%* [NS] (-2.0 - 100)	18%* [NS] (-3.1 - 100)	23%* [NS] (-4.2 - 100)	27%* [NS] (-5.4 - 100)
19p13.12 deletion	3.62	Del	13/ 32587	0/ 269885	100% (31 - 100)	100% (45 - 100)	100% (55 - 100)	100% (62 - 100)	100% (67 - 100)
22q11.2 deletion Velocardiofacial Syndrome and 22q11.2 duplication [*TBX1*] [a]	1.24 or 2.88 *	Del	188/ 33010	10/ 269885	63%* (50 - 80)	75%* (64 - 88)	82%* (73 - 92)	86%* (78 - 94)	89%* (82 - 95)
		Dup	146/ 49060	167/ 269885	4.0%* (3.0 - 5.2)	7.1%* (5.4 - 9.0)	10%* (7.9 - 13)	13%* (10 - 17)	16%* (13 - 20)

22q11.2 distal deletion and 22q11.2 distal duplication [*BCR*, *MAPK1*] [a]	1.75 or 1.82 or 1.87	Del	26/ 23803	1/ 269885	76% (46 - 100)	85% (61 - 100)	90% (70 - 100)	92% (76 - 100)	94% (80 - 100)
		Dup	18/ 23803	7/ 269885	24%* (12 - 50)	36%* (20 - 64)	46%* (27 - 73)	53%* (33 - 78)	59%* (38 - 82)
22q13.33 deletion Phelan-McDermid Syndrome and 22q13.33 duplication [*SHANK3*] [a]	0.06 *	Del	45/ 15767	3/ 269885	74%* [b] (53 - 100)	84%* [b] (68 - 100)	88%* [b] (76 - 100)	91%* [b] (81 - 100)	93%* [b] (84 - 100)
		Dup	*	*	*	*	*	*	*
Xp22.3 duplication [*SHOX*]	0.04-1.3	Dup	83/ 18947	25/ 12594[e]	1.3%[e] (0.5 - 2.8)	2.4%[e] (0.9 - 4.9)	3.5%[e] (1.3 - 7.2)	4.6%[e] (1.7 - 9.4)	5.8%[e] (2.2 - 12)

Supplemental Table 4: This supplemental table is similar to Table 1 in the main text, showing genomic size and penetrance of 83 CNVs from 8 studies (3, 4, 7, 10, 14, 15, 17, 19). Data from affected and control cohorts are the same as that in Table 1, including restriction to individuals with ID where possible. This Supplemental Table provides extra columns for penetrance estimates using the newly proposed formula and values of P(D) ranging from 1.1% to 5.08%. It is not recommended that these penetrance estimates be used in clinical practice because we believe P(D) values higher than 1.1% are not compatible with the input data for the formula. However, some clinicians or researchers may have a niche use for penetrance of a specific phenotype in a specific CNV where a different value of P(D) might be considered more appropriate, hence this supplemental table.

[a]: One or more studies publishing penetrance estimates for this CNV have been excluded, either because it is a duplicate dataset or for risk of bias reasons that are outlined in the systematic review from which this table was adapted (21).

[b]: This CNV is likely to be fully, or nearly fully, penetrant for ID.

**: There are complexities in the genomic coordinates or data used to calculate penetrance for this CNV. The penetrance estimates could be misleading and caution is recommended when interpreting these estimates in a clinical setting. A recent systematic review discussed this in their Supplemental Analysis (21).*

[d]: Genomic coordinates are provided in Supplemental Table 1.

[e]: There is no gnomAD v4.0 CNV data for CNVs on the X chromosome. The original study's control cohort is used instead for penetrance estimation.

[NS]*: This penetrance estimate is not statistically significant. Statistical significance is achieved when the lower 95% confidence interval is greater than 0% using the new penetrance formula. Negative penetrance estimates using the new formula occur when the prevalence of the CNV in control cohorts is greater than that in affected cohorts (Supplemental Methods 1.1).*

Supplemental Results 2 – Prevalence of very low penetrant CNVs in gnomAD v4.0 controls

The gnomAD v4.0 CNV non-neuro controls included 34 CNVs larger than 10Mb. Removal of this led to 269,885 control individuals with 280,951 CNVs, as provided by an excel dataset available in the Supplemental Table of an earlier study (21). Note that this is different from the larger gnomAD v4.0 CNV dataset of 464,297 individuals who have not been marked as controls.

Prevalence of 14 CNVs in this gnomAD CNV control dataset was calculated using the following method. The 14 CNVs were grouped in 3 categories – non-penetrant, negligible penetrance and very low penetrance, as outlined below:

- Category 1 Non-penetrant CNVs: Penetrance is estimated at approximately 0-2% with a 95% confidence interval for penetrance that includes 0%. There is no evidence for pathogenicity of these CNVs when comparing affected cohorts to controls in large scale studies. It is possible that these CNVs may be found to be penetrant in future (larger) datasets, but the current publicly available datasets from the listed studies do not provide evidence for this.
- Category 2 CNVs with negligible penetrance: Penetrance is estimated at 0-2% and the lower 95% confidence interval for penetrance is positive (i.e. statistically significant) but <1%. Care is suggested when assigning pathogenicity to these CNVs since the slight elevation of CNV prevalence in affected compared to control cohorts could be due to other reasons such as publication bias, demographic biases, differences in genetic ancestry, or other study biases.
- Category 3 CNVs with very low penetrance: Penetrance is estimated at 1-3% with the lower 95% confidence interval starting from 1-2%. The discussion section of this study addresses that pathogenicity of these CNVs can be challenging to determine from large scale studies that calculate penetrance, as the minor elevation in penetrance could be due to various biases, or could be due to pathogenicity.

Prevalences in gnomAD v4.0 controls for each of the 7 CNVs in Category 1, the 4 CNVs in Category 2 and the 3 CNVs in Category 3 are provided in the excel spreadsheet Supplemental Table 2.

In gnomAD v4.0 controls, 1.8% of individuals had at least one Category 1 CNV, 0.5% had at least one Category 2 CNV and 0.4% had at least one Category 3 CNV (Supplemental Table 2).

On average, Category 1 CNVs had a prevalence of 0.26%, Category 2 a prevalence of 0.12%, and Category 3 a prevalence of 0.15%, in gnomAD.

In gnomAD v4.0 controls, 2.3% of individuals had at least one Category 1 or 2 CNV.

In gnomAD v4.0 controls, 2.7% of individuals had at least one of these 14 CNVs from Category 1, 2 or 3.

These numbers take into account the possibility of individuals harbouring 2 or more CNVs, as shown in the calculations in Supplemental Table 2.

Supplemental Results 3 – Converting earlier penetrance estimates to new penetrance

A method of converting penetrance estimates from earlier publications to the new penetrance is provided (Supplemental Figure 1). The values for prevalence of disease P(D) = 3-5.3% in the figures, represents the range most commonly chosen for neurodevelopmental disorders in previous studies (3, 4, 7, 10, 14, 15, 17), with a median value of 5.08% (21) used as a comparison.

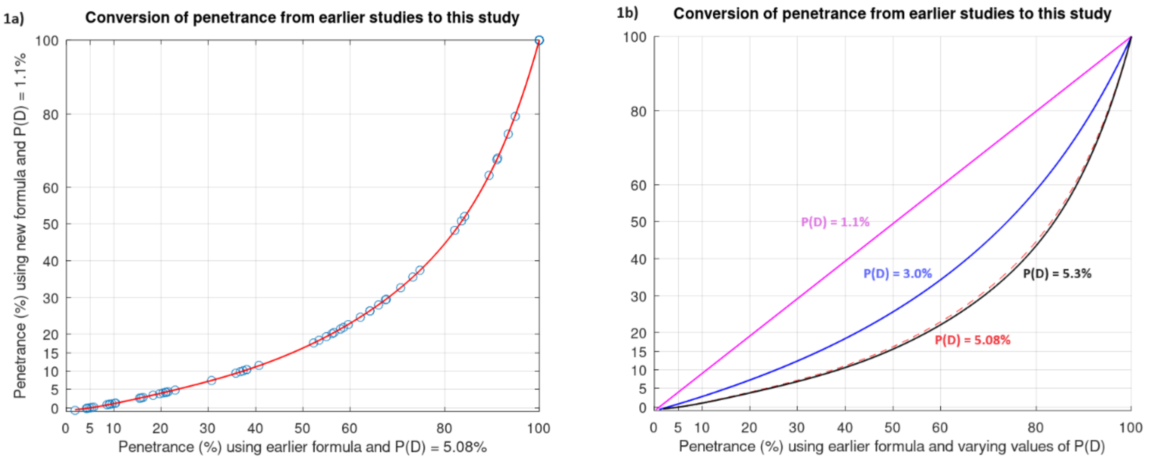

Supplemental Figure 1: a) Penetrance estimates for 83 CNVs in earlier studies (circles) derived using the earlier formula and a disease prevalence P(D) value of 5.08% (x-axis), against penetrance estimates in this study derived using the new formula and a disease prevalence P(D) value of 1.1% (y-axis). b) Figure showing conversion using the earlier penetrance formula and P(D) = 1.1%, 3.0%, 5.08% and 5.3% (x-axis), to the new penetrance formula with P(D) = 1.1% (y-axis). Note that these lines do not go through the origin (0,0); If prevalence of the CNV is equal in affected and control cohorts, this will result in 0% penetrance using the new formula, but result in a penetrance of 1.1%, 3.0%, 5.08% or 5.3% using the earlier formula, depending on the value of disease prevalence, because the earlier formula includes the disease prevalence from the control cohort.

Figure 1a) and 1b) plot the earlier and new penetrance estimates for 83 CNVs and a line of best fit. Earlier penetrance estimates of 10%, 20% and 40% are re-estimated at 1%, 4% and 10% respectively. Fully penetrant conditions remain fully penetrant.

Supplemental Discussion 1 – Various methods of approaching the problem of penetrance using cohorts with multiple phenotypes

Supplemental Discussion 1 explores several other approaches that were considered in attempting to obtain accurate penetrance estimates using these datasets. The final approach adopted by this study is outlined in Supplemental Methods 3 and this section is included for completeness to document the limitations identified using other approaches.

Supplemental Discussion 1.1 – How other studies approached the complexity of multiple phenotypes and limitations associated with these approaches

When the phenotype (or Disease) "D" refers to multiple phenotypes, other studies chose their value of P(D) to be the sum of the prevalence of all these phenotypes. Various studies determined or chose their value of P(D) in one of several different ways:

- Commencing with a high value (e.g. 5.3%) (3) and then providing reasons to subtract from this value until a desired value of P(D) was obtained (14).
- Starting with 0% and then adding conditions (e.g. intellectual disability (1%), autism (1%), schizophrenia (1%), etc), until a desired value of P(D) was obtained (4, 8).
- Citing a previous study's value of P(D), even if their affected cohorts may or may not match (7, 9-12).
- Not explicitly stating their value of P(D) but providing sufficient data for this parameter to be calculated from their published results by using the formula in reverse (17, 19).

Studies that commenced with a value of P(D) = 5.3% base this value on work done in 1988 (48) which counted the prevalence of all known genetic syndromes at the time. Counting each syndrome like this was likely an overestimation due to publication bias, but simultaneously an underestimation since many genetic syndromes were not known at the time of the study in 1988. In addition, P(D) in the formula requires the prevalence of disability, regardless of whether these are genetic in origin or not. A P(D) value of 5.3% is likely an under-representation of the prevalence of disability. However, it is an over-representation of the prevalence of intellectual disability (Supplemental Methods 3.2).

Authors that commenced with a value of P(D) = 0% and build up by adding conditions sometimes do not provide strong evidence for the prevalence of the conditions added. In addition, the prevalence of schizophrenia is likely to be age-dependent, making this value difficult to determine for the purposes of this mathematical formula in studies that used affected cohorts with schizophrenia.

Values of P(D) used in previous studies include 13% (19), 5.3% (3), 5.12% (7, 12, 14), 4% (4, 10, 11, 15), 3% (9), or 1% (8). The difference between studies is stated by their authors to be due to the type of disease "D" that the authors are studying, with studies looking at all types of disability generally reflecting a higher value of P(D). However, some studies with similar definitions of "D" are noted to use different values of P(D). The opposite also holds with some studies reporting different definitions of "D" noted to use similar values of P(D). This discrepancy reflects that there is a somewhat arbitrary choice for authors when choosing the value of P(D) for their study. A choice of 5.3% instead of 3% whilst keeping all other numbers identical in the formula for penetrance will result in almost double the penetrance estimate. Therefore, the choice for this parameter is vital for accurate penetrance estimation.

As a concrete example, consider three studies that used P(D) = 5.12%. One study (7) included individuals with intellectual disability, developmental delay and autism spectrum disorder. Another study (14) with the same value of P(D) included individuals with these same 3 conditions and also those with epilepsy, congenital malformations, dysmorphic features and unspecified issues. A third study focusing on prenatal and neonatal phenotypes (12) counted affected fetuses as those with minor anomalies, including increased nuchal translucency, which is a finding that can resolve during pregnancy and is often compatible with healthy life. These 3 affected cohorts are clearly different, yet all use the same value of P(D). It is difficult to compare these studies further, since they studied different CNVs. However, the 3 studies serve as an excellent example of how a value of P(D) = 5.12% used in one study (14) can be used again in other studies with different affected cohorts, even when the 3 studies should use 3 different values of P(D). In all 3 cases, a lower level of P(D) is probably more accurate (Supplemental Methods 3.2).

Supplemental Discussion 1.2 – A weighted value of P(D) for penetrance of multiple phenotypes

The penetrance formula is ideally suited to explore penetrance for a single phenotype (Supplemental Methods 3.1). Using affected cohorts with multiple phenotypes and choosing a value of P(D) that represents the sum of the prevalence of multiple phenotypes to derive a penetrance estimate (Supplemental Discussion 1.1) is inappropriate because ID is the dominant phenotype in the affected cohort (Supplemental Methods 3.2). This section explores the possibility of overcoming this limitation by using a weighted average of P(D) in the formula to represent different phenotypes. The conclusion is that this is not as robust an approach as using P(D) = 1.1%.

A weighted average between P(D) = 1.1% for ID and P(D) = 5.3% for multiple miscellaneous phenotypes (3) is tempting to consider if the cohort is primarily that of ID but has a few individuals with other phenotypes. In such a scenario, the final P(D) value would be somewhere between 1.1% and 5.3%, but weighted closer to 1.1% given the affected cohorts are composed primarily of individuals with ID (Supplemental Methods 3.2). We show why this is not an optimal choice by exploring 3 scenarios:

1. For a CNV which is not strongly associated with a physical (non-ID) phenotype, choosing a value of P(D) above 1.1% is clearly unhelpful.
2. For a CNV which is partially associated with a physical phenotype and partially associated with ID, then the only individuals unaccounted for are those with the physical phenotype but without ID. In such a situation, it is unclear how many more percentage points to add for P(D) on top of its default of 1.1%, nor is it clear that this physical phenotype was sampled in the affected cohort a manner proportionate to those with ID. This makes any choice of P(D) greater than 1.1% somewhat arbitrary.
3. For CNVs that are strongly associated with a physical phenotype and not associated with ID (e.g. 17q12 [*HNF1B*] deletion which causes renal cysts and diabetes), a P(D) value of 1.1% (to represent the prevalence of ID of 1.1%) is clearly inaccurate. The prevalence of renal cysts in childhood is 0.2-2% (49) and that of diabetes is approximately 0.2% (50). If these two figures are added together and a small subtraction undertaken to account for individuals who might have been double counted if they had both renal cysts and diabetes, then the prevalence of

either diabetes or renal cysts might be around 0.3% in children (for instance). A P(D) value of 0.3% could be considered for use in the formula. However, the data used for P(G|D) is a cohort of individuals primarily with intellectual disability, which is not the phenotype of the 17q12 [*HNF1B*] deletion. Therefore, this data cannot be used to estimate penetrance for the 17q12 [HNF1B] deletion, even if an appropriate value of P(D) can been determined. This is why penetrance estimates for CNVs with a well described phenotype and no evidence for ID (or CNVs where ID is not the most common phenotype) are not included in Table 1.

In summary, if the CNV is strongly associated with a physical but not an intellectual phenotype, then the data from these studies (3, 4, 7, 10, 14, 15, 17, 19) cannot be used because the data includes individuals who primarily have ID (which is irrelevant for the CNV). If the CNV is partially associated with a physical phenotype and ID, it remains unclear if the sampling of this phenotype of individuals without ID and sampling of individuals with ID was equally homogenous, making a choice of P(D) greater than 1.1% somewhat arbitrary. Therefore, this study was unable to determine a feasible way to perform a weighted average of phenotypes in the affected cohort to determine an optimal value of P(D). A choice of 1.1% may already be optimal in most cases.

Supplemental Discussion 1.3 – Conclusion regarding the optimal value of P(D)

Supplemental Methods 3.2 demonstrate that the majority of individuals in the affected cohorts used in this study (3, 4, 7, 10, 14, 15, 17, 19) have ID. Supplemental Methods 3.3 shows that the prevalence of ID, P(D), is approximately 1.1%. Supplemental Discussions 1.1 & 1.2 investigate alternate values for P(D) and establish that none of these are superior to the 1.1% estimate.

Although there is no perfect value that P(D) can take given that the datasets for affected cohorts are imperfect, the most accurate interpretation of the currently available data using a Bayesian formula for penetrance requires that the parameter P(D) in the formula for penetrance be approximately 1.1%.

Supplemental Discussion 2 – 95% confidence intervals for penetrance estimates

Penetrance estimates derived from the formula using data from existing datasets, should be thought of as potentially lying within a range of possible values, rather than as a fixed percentage.

For example, the penetrance for ID for the 16p13.3 deletion (Rubinstein-Taybi Syndrome) [*CREBBP*] was estimated in this study to be 47% (95% confidence interval 17-100%). This estimate of 47% is likely too low and is due primarily to the single individual present in the control cohort (1/269,885). The small affected cohort (10/32,587) is also partially responsible for this (erroneously) low penetrance estimate.

It should be noted that the single individual in the control arm with a 16p13.3 deletion may be mosaic for the condition, have a somatic proliferation in blood for this deletion, be due to a contaminated sample, pre-analytic error (wrong patient label on collected blood or wrong sample), analytic error (machine error, data entry error), have mild symptoms due to variable expressivity of the condition, or might have classic Rubinstein-Taybi syndrome and was erroneously included in a control cohort due to lack of proper documentation or lack of proper phenotyping by the study.

The true penetrance estimate of the 16p13.3 deletion, based on case series, is probably closer to 100% (51). It is therefore more accurate to say that the data suggests that the penetrance for ID in this condition is, with 95% confidence, likely to be between 17% to 100%.

Supplemental References

1. Carruth ED, Young W, Beer D, James CA, Calkins H, Jing L, et al. Prevalence and Electronic Health Record-Based Phenotype of Loss-of-Function Genetic Variants in Arrhythmogenic Right Ventricular Cardiomyopathy-Associated Genes. Circ Genom Precis Med. 2019;12(11):e002579.
2. Chen S, Parmigiani G. Meta-analysis of BRCA1 and BRCA2 penetrance. J Clin Oncol. 2007;25(11):1329-33.
3. Cooper DN, Krawczak M, Polychronakos C, Tyler-Smith C, Kehrer-Sawatzki H. Where genotype is not predictive of phenotype: Towards an understanding of the molecular basis of reduced penetrance in human inherited disease. Hum Genet. 2013;132(10):1077-130.
4. Kirov G, Rees E, Walters JT, Escott-Price V, Georgieva L, Richards AL, et al. The penetrance of copy number variations for schizophrenia and developmental delay. Biol Psychiatry. 2014;75(5):378-85.
5. Vassos E, Collier DA, Holden S, Patch C, Rujescu D, St. Clair D, et al. Penetrance for copy number variants associated with schizophrenia. Hum Mol Genet. 2010;19(17):3477-81.
6. Roberts JD, Asaki SY, Mazzanti A, Bos JM, Tuleta I, Muir AR, et al. An International Multicenter Evaluation of Type 5 Long QT Syndrome: A Low Penetrant Primary Arrhythmic Condition. Circulation. 2020;141(6):429-39.
7. Allach El Khattabi L, Heide S, Caberg JH, Andrieux J, Doco Fenzy M, Vincent-Delorme C, et al. 16p13.11 microduplication in 45 new patients: refined clinical significance and genotype-phenotype correlations. J Med Genet. 2018;0:1-7.
8. Chaste P, Sanders SJ, Mohan KN, Klei L, Song Y, Murtha MT, et al. Modest impact on risk for autism spectrum disorder of rare copy number variants at 15q11.2, specifically breakpoints 1 to 2. Autism Res. 2014;7(3):355-62.
9. Cosemans N, Vandenhove L, Vogels A, Devriendt K, Van Esch H, Van Buggenhout G, et al. The clinical relevance of intragenic NRXN1 deletions. J Med Genet. 2020;57(5):347-55.
10. Isles AR, Ingason A, Lowther C, Walters J, Gawlick M, Stober G, et al. Parental Origin of Interstitial Duplications at 15q11.2-q13.3 in Schizophrenia and Neurodevelopmental Disorders. PLoS Genet. 2016;12(5).
11. Kendall KM, Bracher-Smith M, Fitzpatrick H, Lynham A, Rees E, Escott-Price V, et al. Cognitive performance and functional outcomes of carriers of pathogenic copy number variants: analysis of the UK Biobank. Br J Psychiatry. 2019;214(5):297-304.
12. Maya I, Perlman S, Shohat M, Kahana S, Yacobson S, Tenne T, et al. Should We Report 15q11.2 BP1-BP2 Deletions and Duplications in the Prenatal Setting? J. 2020;9(8):11.
13. Mohan KN, Cao Y, Pham J, Cheung SW, Hoffner L, Ou ZZ, et al. Phenotypic association of 15q11.2 CNVs of the region of breakpoints 1-2 (BP1-BP2) in a large cohort of samples referred for genetic diagnosis. J Hum Genet. 2019;64(3):253-5.
14. Rosenfeld JA, Coe BP, Eichler EE, Cuckle H, Shaffer LG. Estimates of penetrance for recurrent pathogenic copy-number variations. Genet Med. 2013;15(6):478-81.
15. Tropeano M, Howley D, Gazzellone MJ, Wilson CE, Ahn JW, Stavropoulos DJ, et al. Microduplications at the pseudoautosomal SHOX locus in autism spectrum disorders and related neurodevelopmental conditions. J Med Genet. 2016;53(8):536-47.
16. Al Shehhi M, Forman EB, Fitzgerald JE, McInerney V, Krawczyk J, Shen S, et al. NRXN1 deletion syndrome; phenotypic and penetrance data from 34 families. Eur J Med Genet. 2019;62(3):204-9.
17. Jønch AE, Douard E, Moreau C, Van Dijck A, Passeggeri M, Kooy F, et al. Estimating the effect size of the 15Q11.2 BP1-BP2 deletion and its contribution to neurodevelopmental symptoms: recommendations for practice. J Med Genet. 2019;56(10):701-10.
18. Unique. Rare Chromosome & Gene Disorder Guides: Rare Chromosome Disorder Support Group; 2024 [Available from: https://rarechromo.org/disorder-guides/.

19. Martin CL, Wain KE, Oetjens MT, Tolwinski K, Palen E, Hare-Harris A, et al. Identification of Neuropsychiatric Copy Number Variants in a Health Care System Population. JAMA Psychiatry. 2020;77(12):1276-85.
20. Maulik PK, Mascarenhas MN, Mathers CD, Dua T, Saxena S. Prevalence of intellectual disability: a meta-analysis of population-based studies. Res Dev Disabil. 2011;32(2):419-36.
21. Goh S, Thiyagarajan L, Dudding-Byth T, Pinese M, Kirk EP. A systematic review and pooled analysis of penetrance estimates of copy-number variants associated with neurodevelopment. Genet Med. 2025;27(1):101227.
22. van Paassen BW, van der Kooi AJ, van Spaendonck-Zwarts KY, Verhamme C, Baas F, de Visser M. PMP22 related neuropathies: Charcot-Marie-Tooth disease type 1A and Hereditary Neuropathy with liability to Pressure Palsies. Orphanet J Rare Dis. 2014;9:38.
23. Chrestian N. Hereditary Neuropathy with Liability to Pressure Palsies. In: Adam MP, Feldman J, Mirzaa GM, Pagon RA, Wallace SE, Bean LJH, et al., editors. GeneReviews(®). Seattle (WA)1993.
24. Benn PA. Prenatal counseling and the detection of copy-number variants. Genet Med. 2013;15(4):316-7.
25. Schultz LM, Knighton A, Huguet G, Saci Z, Jean-Louis M, Mollon J, et al. Copy-number variants differ in frequency across genetic ancestry groups. HGG Adv. 2024;5(4):100340.
26. Muys J, Blaumeiser B, Jacquemyn Y, Bandelier C, Brison N, Bulk S, et al. The Belgian MicroArray Prenatal (BEMAPRE) database: A systematic nationwide repository of fetal genomic aberrations. Prenat Diagn. 2018;38(13):1120-8.
27. Zhou D, Gochman P, Broadnax DD, Rapoport JL, Ahn K. 15q13.3 duplication in two patients with childhood-onset schizophrenia. Am J Med Genet B Neuropsychiatr Genet. 2016;171(6):777-83.
28. Gillentine MA, Schaaf CP. The human clinical phenotypes of altered CHRNA7 copy number. Biochem Pharmacol. 2015;97(4):352-62.
29. ClinGen. 15q13.3 recurrent region (D-CHRNA7 to BP5) (includes CHRNA7 and OTUD7A): ClinGen Dosage Sensitivity Curation Page; 2018 [updated 10/5/2018. Available from: https://dosage.clinicalgenome.org/clingen_region.cgi?id=ISCA-46295.
30. Cai M, Que Y, Chen X, Chen Y, Liang B, Huang H, et al. 16p13.11 microdeletion/microduplication in fetuses: investigation of associated ultrasound phenotypes, genetic anomalies, and pregnancy outcome follow-up. BMC pregnancy and childbirth. 2022;22(1):913.
31. Muys J, Jacquemyn Y, Blaumeiser B, Bourlard L, Brison N, Bulk S, et al. Prenatally detected copy number variants in a national cohort: A postnatal follow-up study. Prenat Diagn. 2020;40(10):1272-83.
32. Smajlagić D, Lavrichenko K, Berland S, Helgeland Ø, Knudsen GP, Vaudel M, et al. Population prevalence and inheritance pattern of recurrent CNVs associated with neurodevelopmental disorders in 12,252 newborns and their parents. Eur J Hum Genet. 2021;29(1):205-15.
33. Fu J, Liao C, Collins R, Wang L, Ben-Isvy D, Brand H, et al. Rare coding CNVs from exome sequenced individuals in gnomAD v4 GnomAD: GnomAD; 2023 [updated 1/11/2023. Available from: https://gnomad.broadinstitute.org/news/2023-11-v4-copy-number-variants/.
34. Babadi M, Fu JM, Lee SK, Smirnov AN, Gauthier LD, Walker M, et al. GATK-gCNV enables the discovery of rare copy number variants from exome sequencing data. Nat Genet. 2023;55(9):1589-97.
35. Shimelis H, Oetjens MT, Walsh LK, Wain KE, Znidarsic M, Myers SM, et al. Prevalence and Penetrance of Rare Pathogenic Variants in Neurodevelopmental Psychiatric Genes in a Health Care System Population. The American journal of psychiatry. 2023;180(1):65-72.
36. BroadInstitute. gnomAD Browser 2023 [Available from: https://gnomad.broadinstitute.org/.
37. Dittwald P, Gambin T, Szafranski P, Li J, Amato S, Divon MY, et al. NAHR-mediated copy-number variants in a clinical population: mechanistic insights into both genomic disorders and Mendelizing traits. Genome Res. 2013;23(9):1395-409.

38. Ahn JW, Dixit A, Johnston C, Ogilvie CM, Collier DA, Curran S, et al. BBGRE: brain and body genetic resource exchange. Database (Oxford). 2013.
39. Howlin P. Prognosis in autism: do specialist treatments affect long-term outcome? Eur Child Adolesc Psychiatry. 1997;6(2):55-72.
40. Fombonne E. Epidemiological surveys of autism and other pervasive developmental disorders: an update. J Autism Dev Disord. 2003;33(4):365-82.
41. Wright B, Spikins P, Pearson H. Should Autism Spectrum Conditions Be Characterised in a More Positive Way in Our Modern World? Medicina (Kaunas). 2020;56(5).
42. Fischbach GD, Lord C. The Simons Simplex Collection: a resource for identification of autism genetic risk factors. Neuron. 2010;68(2):192-5.
43. Zeidan J, Fombonne E, Scorah J, Ibrahim A, Durkin MS, Saxena S, et al. Global prevalence of autism: A systematic review update. Autism Res. 2022.
44. Lai MC. Editorial: Meaningfully Stratifying the Autism Spectra. J Am Acad Child Adolesc Psychiatry. 2020;59(12):1324-6.
45. Rosen NE, Lord C, Volkmar FR. The Diagnosis of Autism: From Kanner to DSM-III to DSM-5 and Beyond. J Autism Dev Disord. 2021;51(12):4253-70.
46. Thabtah F, Peebles D. Early Autism Screening: A Comprehensive Review. Int J Environ Res Public Health. 2019;16(18).
47. Auwerx C, Lepamets M, Sadler MC, Patxot M, Stojanov M, Baud D, et al. The individual and global impact of copy-number variants on complex human traits. Am J Hum Genet. 2022;109(4):647-68.
48. Baird PA, Anderson TW, Newcombe HB, Lowry RB. Genetic disorders in children and young adults: a population study. Am J Hum Genet. 1988;42(5):677-93.
49. Ferro F, Vezzali N, Comploj E, Pedron E, Di Serafino M, Esposito F, et al. Pediatric cystic diseases of the kidney. J Ultrasound. 2019;22(3):381-93.
50. Chen Y, Wang T, Liu X, Shankar RR. Prevalence of type 1 and type 2 diabetes among US pediatric population in the MarketScan Multi-State Database, 2002 to 2016. Pediatr Diabetes. 2019;20(5):523-9.
51. van Belzen M, Bartsch O, Lacombe D, Peters DJ, Hennekam RC. Rubinstein-Taybi syndrome (CREBBP, EP300). Eur J Hum Genet. 2011;19(1):preceeding 118-20.
